# Closed-loop high-precision two-photon lithography based on a multiplexed single-cavity dual-comb laser

**DOI:** 10.1038/s41467-026-73972-7

**Published:** 2026-06-03

**Authors:** Yalan Yu, Zhiwei Zhu, Benjamin Willenberg, Justinas Pupeikis, Christopher R. Phillips, Shih-Chi Chen

**Affiliations:** 1 Department of Mechanical and Automation Engineering, The Chinese University of Hong Kong, Shatin, N.T. Hong Kong; 2 Department of Physics, Institute for Quantum Electronics, ETH, Zurich, Switzerland; 3 Centre for Perceptual and Interactive Intelligence, Hong Kong Science Park, Shatin, N.T. Hong Kong

**Keywords:** Engineering, Mechanical engineering

## Abstract

Two-photon lithography fabricates three-dimensional structures with 100-nanometer resolution; yet its industrial adoption is hindered by poor reproducibility and the need for complex manual tuning processes. While post-fabrication metrology, such as scanning electron microscopy, characterizes final morphologies, it cannot prevent manufacturing errors. Here we show a two-photon lithography platform powered by a single-cavity dual-comb laser that addresses this limitation through real-time correction. During fabrication, one laser comb, after frequency conversion, performs two-photon printing, while the dual-comb system simultaneously performs in-situ phase measurements across the full work field at 360 hertz. By feeding the phase profiles into a dynamic model, the platform automatically modulates printing parameters to correct height errors. We demonstrate this capability through a continuous 14-hour fabrication process to manufacture different millimeter-scale diffractive optical elements with less than 100-nm absolute errors. The resulting devices exhibit superior signal-to-noise ratios, process repeatability, and focus quality. This closed-loop dual-comb platform offers a cost-effective, scalable solution for high-precision, high-yield nanomanufacturing.

## Abstract (web summary)

A closed-loop two-photon printing system based on a single-cavity dual-comb laser is developed to eliminate traditional complex calibration processes and significantly improve the fabrication reproducibility for largescale continuous nanofabrication.

## Introduction

Two-photon lithography (TPL) has emerged as one of the most widely used micro-additive manufacturing methods for creating nanoscale three-dimensional (3D) structures and devices in recent years^1,2^. Optical components or devices, such as metastructures and diffractive optical elements (DOEs), offer an enticing prospect for applications in the fields of virtual reality/augmented reality^3^, optical network computing^4^, pulse shaping^5,6^, biomedical imaging^7^, and chemical sensing^8^, etc. To practically apply the TPL technology for producing large-scale devices, besides improving the fabrication rate and cost reduction^9,10^, one still needs to achieve high reproducibility, i.e., to consistently fabricate precise morphological dimensions at ~200 nm over a long period of time (>10 hours) during the TPL printing process.

Although most high-resolution photo-polymerization systems would include a custom-built microscopic imaging module for monitoring the process in situ^1,2,9–11^, it does not directly improve the printing results because such imaging modules do not offer 3D imaging capability and are limited by diffraction, i.e., one cannot observe features at 200 nm or below. Characterization of TPL printed parts is mostly performed post-fabrication via scanning electron microscopy (SEM) or atomic force microscopy (AFM), which provides nanometer-scale morphological information. It is worth noting that these methods cannot be integrated with TPL for in-situ process control owing to incompatibility with the liquid-based printing process. On the other hand, a few imaging methods, including quantitative phase imaging^12^, optical coherence tomography^13^, and optical diffraction tomography^14,15^, may be integrated with TPL to in-situ monitor the 3D printing process through the variation of refractive index (RI) distribution in the work field. However, all reported methods for monitoring TPL to date used an open-loop strategy, i.e., the TPL system used pre-defined printing parameters throughout the fabrication process, and could not correct morphological deviations caused by inevitable process parameter fluctuations (including laser power, vibration, etc.) in real time, leading to errors ranging from inaccurate part height to inconsistent part dimensions. Although such fabrication errors are negligible for small-scale structures with a fine-calibrated TPL system, they will increase continuously over time and eventually degrade the quality of large-scale structures or devices. Thus, the reproducibility issue is more pronounced for long fabrication sessions or printing of large parts, where maintaining constant process parameters becomes challenging. Conventionally, the solution to this challenge is largely empirical: repeating the fabrication process multiple times with more controlled process parameters or environmental conditions to generate a qualified part. This is not only time-consuming and expensive but also environmentally damaging. A precise and robust closed-loop control method for TPL to improve its quality and reproducibility has yet to be developed.

Here, we present a closed-loop TPL platform based on a multiplexed single-cavity dual-comb laser. In the system, the first comb is used to perform continuous TPL at 800nm (90% power). Simultaneously, the dual-comb-based system performs in-situ phase measurement in photoresists at 1052 nm via a spectrum-encoding technique^16^ over the entire work field, generating instantaneous phase and height information at ~360 Hz. The measured phase information is fed to a dynamic model that modulates the laser power, scanning rate, and exposure time at 1Hz to correct fabrication errors and substantially improve long-term printing stability and part reproducibility. Our experiments show that the closed-loop TPL achieves an in-situ height measurement resolution of 4.8 nm and a lateral imaging resolution of 1.10 µm. To demonstrate the TPL system, we designed and fabricated a millimeter-scale 11-level DOEs and 8-level phase-type Fresnel zone plate (FZP) lenses. Imaging experiments were devised and performed based on the fabricated DOEs and FZP lenses. The results demonstrated substantially improved signal-to-noise ratio and focus quality, affirming that the DOEs and FZP lenses were fabricated with less than 100-nm fabrication tolerances. With high reproducibility, the closed-loop TPL is ready to produce large-scale optical parts for various practical applications.

## Results

### Design of the closed-loop two-photon lithography system

**Fig. 1:**
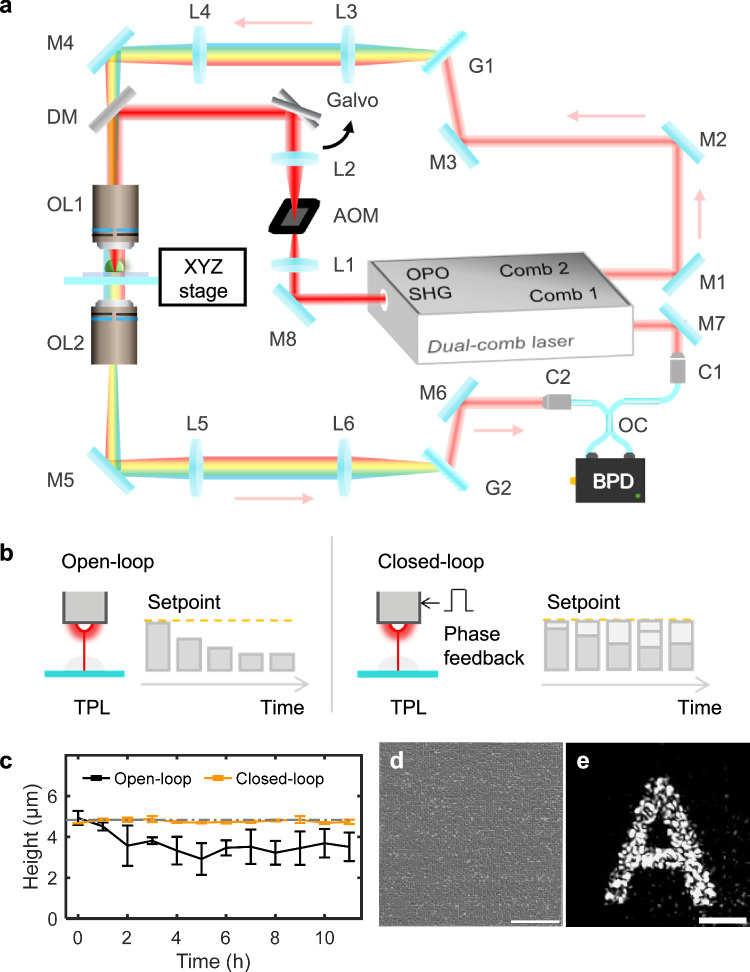
Configuration and principle of the closed-loop two-photon lithography system. **a** Optical configuration of the closed-loop two-photon lithography system based on a single-cavity dual-comb laser. PBS: polarization beam splitter; BS: beam splitter; G: transmission diffraction grating; OL: objective; M1-M8: high reflectivity mirrors; DM: dichroic mirror; C: fiber collimator; OC: fiber optic coupler; BPD: balanced photodetector; AOM: acousto-optic modulator; Galvo: galvanometric scanners; L1-L6: lenses (focal lengths: 100, 200, 60, 150, 60, 150 mm, respectively). **b** Target dimension (e.g., heights) can be precisely maintained over long printing sessions in a closed-loop two-photon lithography system in comparison to an open-loop two-photon lithography system. TPL: two-photon lithography. **c** Comparison of measured heights in open-loop (gray) and closed-loop (orange) systems. The dashed line denotes the target height (4.8 µm) in experiments. Error bars represent the standard deviations for height measurements. **d** Scanning electron microscopy image of the closed-loop written diffractive optical element. Scale bar: 200 μm. **e** Measured diffraction pattern of the diffractive optical element. Scale bar: 200 μm.

Figure 1a presents the optical configuration of the closed-loop TPL system. The light source is a custom-built single-cavity dual-comb laser (1052 nm, 140 fs, 2 W, 80 MHz)^17^ that enables simultaneous TPL writing and in-situ phase imaging for closed-loop control. For phase imaging, the repetition rate difference of the two combs is set to 356 Hz and stabilized via phase-locked loops to improve signal-to-noise ratio and image quality for long-term operations. For TPL, 800-nm femtosecond laser pulses are generated via a custom-built wavelength conversion unit, which downconverts Comb1 (1.8 W) to 1600 nm via a periodically poled MgO:LiNbO_3_ crystal; and subsequently upconverts the output beam to 800 nm via the second harmonic generation (not shown in Fig. 1). The laser pulses for TPL have a measured center wavelength of 800 nm, average power of 290 mW and pulse duration of 151 fs.

To perform TPL, the 800-nm beam first passes through an acousto-optic modulator (AOM, MLP210-1DC, Gooch & Housego), which serves as a fast optical switch (210 MHz), and then relayed to a two-axis galvanometer scanner (Galvo, QS20XY-AG, Thorlabs, Inc.), which are synchronized to perform in-plane raster scanning. After the dichroic mirror (DM), the scanning beam is focused by an oil-immersion objective lens (OL1, CFI S Plan Fluor ELWD 40×, NA 0.6, Nikon) to write designed structures in photoresists (i.e., IPL-780). The sample is mounted on a precision XYZ stage for continuous large-scale printing. To measure the phase change of the laser-exposed photoresists during TPL, Comb2 first passes through a transmission diffraction grating (G1, T-1000-1040s, LightSmyth), where the dispersed laser beam is expanded via a 4-f system (L3 and L4); after a high reflectivity mirror (M4) and the DM, Comb2 is focused to the build plane via OL1 to form a line illumination. The transmitted light is collected by a second objective (OL2, CFI S Plan Fluor ELWD 40×, NA 0.6, Nikon). After a mirror M5, lenses (L5, L6), and a grating (G2, T-1000-1040s, LightSmyth), the laser pulses are recompressed and enter a 50:50 fiber-optic coupler (OC) to interfere with Comb1 (i.e., unmodulated signals). Lastly, the interfered signals are collected by a balanced differential detector (BPD, PDB415C-AC, Thorlabs, Inc.) and digitized via a data acquisition board with a sampling rate of 500 MS/s. The in-situ phase imaging capability enables close-loop TPL operation, which greatly enhances the part reproducibility. (Note that the phase imaging setup can also be configured in a reflective mode with the same performance; details are provided in the Supplementary Methods and Supplementary Fig. 1).

Figure 1b illustrates the working principle of the closed-loop TPL system, where accurate morphologies (e.g., height) of the laser-exposed parts are continuously extracted from the measured phase images; accordingly, the system can suitably adjust the laser exposure time to compensate for the measured dimensional deviations, achieving the target dimension (i.e., setpoint) within the set threshold (i.e., allowable errors). Although TPL is known to have high printing precision, it has always been operated in an open-loop fashion; thus, during a long printing session (e.g., > 10 h), part dimension varies unavoidably up to micrometer level. Figure 1c compares closed-loop and open-loop TPL writing results of an array of cuboids (8.0 µm × 8.0 µm× 4.8 µm in size) over 10 hours. (The experiments were repeated three times.) The results show the closed-loop system maintains accurate heights throughout the long printing session. (Average errors for closed-loop and open-loop heights are 33 nm and 1139 nm, respectively.) With precise height control, we fabricated a large-scale DOE (790 µm × 790 µm) (Fig. 1d) to demonstrate the improved contrast and imaging results (Fig. 1e).

### Realization of in-situ nanoscale morphology measurements

**Fig. 2:**
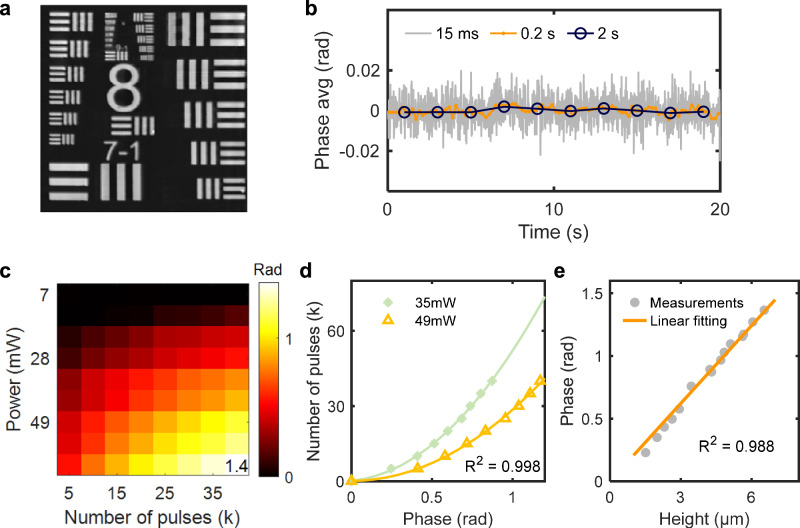
Measurement of phase differences and part heights. **a** Retrieved phase image of the standard 1951 United States Air Force resolution test chart. **b** Measured temporal phases with different averaging times. **c** In situ measured phase differences of 9×8 slabs using different laser processing parameters. **d** Representative relation between the measured phase differences and laser processing parameters, including the number of pulses and power. **e** Relation between the measured phase differences and part heights, measured via atomic force microscopy.

We used a standard 1951 United States Air Force (USAF) resolution target to characterize the spectrum-encoding phase imaging results, where the target was placed at the focal plane of OL1. Figure 2a shows the retrieved phase image of the resolution target (element 6 of group 9), which has a lateral resolution of 1.10 µm. The phase sensitivity (i.e., standard deviations (s.d.) of the measured phase changes induced by curing) of our system was assessed by successively measuring the printed cuboids in the photoresist. Figure 2b presents the phase measurements (subtracting the mean value) with different averaging times, where the s.d. are 0.065 rad for 15 ms, 0.002 rad for 0.2 sec, and 0.001 rad for 2 sec. To investigate the effective phase difference in the photoresist, we printed an 9 × 8 array of single-layer slabs (4.7 µm × 11.9 µm) using nine different laser powers (7–63 mW in steps of 7 mW) and eight different laser pulses (5,000 – 40,000 in steps of 5000) at a scanning speed of 30 mm/sec; and a voxel distance of 100 nm. (Note that the number of laser pulses here refers to the average pulses received per curing voxel during scanning, which is mainly set by the AOM.) The in-situ measured phase differences (in reflective mode) are presented in Fig. 2c, which show phase differences ranging from 0.01 rad to 1.41 rad. This indicates that our system can fabricate structures with various heights and in situ measure precise phase differences. Figure 2d presents the relationship between the measured phase differences and laser processing parameters, including the number of pulses and power. (Here, the data can be precisely fitted by a polynomial function, which will be used for closed-loop laser parameter control in the next section.) To correlate the measured phase differences with the part heights, the heights of the printed structures were measured by an AFM. The results are presented in Fig. 2e. Specifically, the measured part heights ($h$) have a quasi-linear relation with the phase differences ($\varDelta \varphi$), i.e., $\varDelta \varphi \cong 0.207{{{\rm{rad}}}}/{{{\rm{\mu }}}}{{{\rm{m}}}}\cdot h$. Notably, after photopolymerization, the RI difference ($\varDelta n$) of the photoresist (i.e., IPL-780; $n\approx 1.504$) is calculated to be 0.0347 for the dual-comb system at 1052 nm^18,19^. (See details in the Methods section.) Based on the results, the height sensitivity in Fig. 2b for 15 ms, 0.2 sec and 2 sec are calculated to be 309.5, 9.5, and 4.8 nm, respectively.

**Fig. 3:**
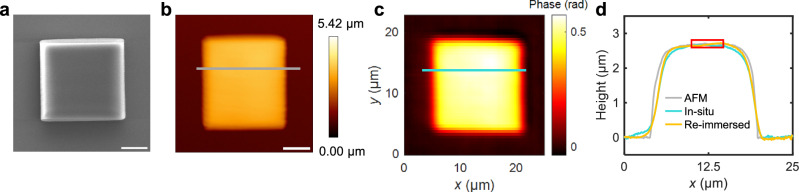
Characterization of part height before and after development. **a–c** Scanning electron microscopy image, atomic force microscopy image, and in-situ phase image during two-photon lithography of a 15 µm × 15 µm single-layer slab, respectively. **d** Comparison of slab height profiles measured by the dual-comb system before and after development (cyan and yellow lines) as well as atomic force microscopy (AFM) (gray lines in **b**). Scale bars: 5 μm (**a,** **b**).

Next, we characterize the volume change between the laser-written part (in the photoresists) and the final part (after development). This is important as the closed-loop control system only measures phase variation in situ during the printing process. (Note that the volume change during the photopolymerization process is captured by the dual-comb system.) Here, we printed a single-layer slab (15 µm × 15 µm) on a glass substrate using an average laser power of 45 mW and a laser scanning speed of 30 mm/s. To compare the slab height immediately after laser exposure and post development, during two-photon printing, we performed in-situ phase measurements on the printed structure and extracted the phase profiles with an averaging time of 47 ms (Fig. 3c) (Note that the 2D phase map was obtained by scanning the focal line (Comb2) in a direction orthogonal to the line by the XYZ stage.) After development, the SEM image shows that the post-processed part closely matches with the design with minimal distortion (Fig. 3a). To characterize the structure shrinkage, we converted the measured phase profiles into height images (See details in the “Methods” section) and compared those with the AFM results. Lastly, we measured the phase profile again by re-immersing the slab in the photoresist. Figure 3d compares the measured slab profiles. The results show that the mean absolute difference (m.s.e.) between the dual-comb measurements and AFM results in the center region of the slab (denoted by the red box) is 14.19 nm, which confirms that the dual-comb system can precisely and rapidly measure the part morphology in situ for closed-loop operations. Notably, the small deviation between the dual-comb measurements (before and after development) reveals that the shrinkage under our processing parameters and conditions is negligible. (Note that negligible or consistent post-development volume shrinkage is a required condition for using our method. This issue can be largely addressed by adjusting the printing power to a moderately high value within the reaction window.)

### Enhancement of reproducibility via closed-loop two-photon lithography

**Fig. 4:**
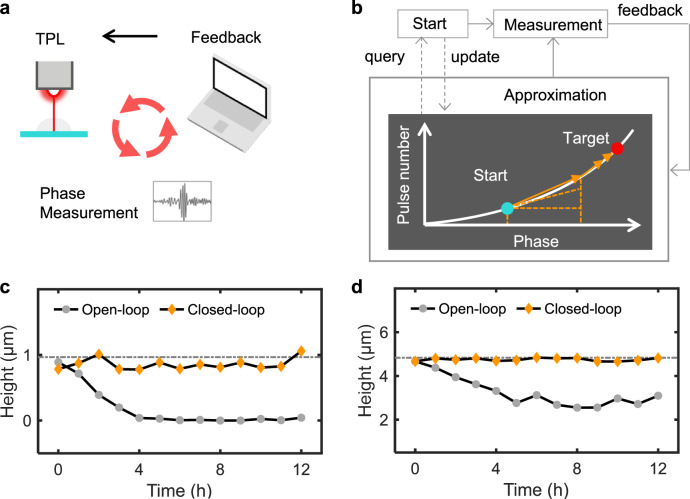
Comparison of open-loop and closed-loop two-photon lithography fabrication. **a** Schematic of closed-loop two-photon lithography with real-time phase measurement via the dual-comb module. TPL: two-photon lithography. **b** Application of the gradient linear approximation method to the dynamic model to approach the target value in closed-loop operation. **c**, **d** Measured heights of the printed cuboids via open-loop (gray) and closed-loop (orange) two-photon lithography systems with target heights of 1.0 μm and 4.8 μm, respectively.

Closed-loop TPL is important as it ensures consistent part dimensions and improves reproducibility over long TPL printing sessions ( > 10 h), which is not uncommon for printing large parts. Figure 4a illustrates how closed-loop TPL is implemented by using the measured phases as feedback signals. By rapidly recording the structure-phase profile, a dynamic model (i.e., the polynomial function in Fig. 2d) that links the printing parameters (i.e., the number of laser pulses) and the measured part dimension can be established to compensate for printing errors over time until the part dimension reaches the set value. To begin, we estimate the required laser pulses using the dynamic model and the first-order gradient linear approximation method (Fig. 4b). Since curing is irreversible, we must ensure the initial laser dose is lower than the setpoint (e.g., a specific part height). As such, we start by using half the predicted dose (i.e., number of laser pulses at a constant laser power). Notably, the measured phase differences are used to update the dynamic model at every new focus position to ensure the model accuracy. The setpoint is then reached by gradually increasing the laser pulses, as mathematically expressed in Eq. (4) (See details in the “Methods” section.).

Next, we compare the printing stability between the open-loop and the closed-loop TPL system. In the experiments, we continuously printed arrays of cuboids with two different target heights (i.e., 8.0 µm × 8.0 µm × 1.0 µm and 8.0 µm × 8.0 µm × 4.8 µm) over 12 h. The precision of the height would, for example, represent the quality of a DOE, as the height of each pixel induces a designed phase delay. During the printing process, the laser was set to a constant power of 45 mW and raster-scanned to fabricate the cuboids at the 30 mm/sec (hatching distance: 100 nm). For the closed-loop system, the dual-comb module monitored the process in situ with a data acquisition time of 47 ms. During open-loop printing, the dual-comb module measured the profiles of the cuboids every hour. Figure 4c, d present the open-loop and closed-loop TPL fabrication results with a target height of 1.0 µm and 4.8 µm, respectively, where one may observe that in both cases the heights of the open-loop printed cuboids started to deviate from the setpoint within 1 hour, while the closed-loop printed cuboids remain highly accurate throughout the entire fabrication processes (Supplementary Fig. 7). Specifically, for the closed-loop results, the average height errors of the 1.0-µm and 4.8-µm tall cuboids are 0.130 µm (s.d. = 0.088 µm) and 0.084 µm (s.d. = 0.069 µm). These results verified that the reproducibility has been substantially improved through our closed-loop fabrication strategy. Regarding the open-loop fabrication results, possible factors that cause dimensional variations include fluctuations in fabrication and environmental parameters, such as drifting laser power, temperature, mechanical vibration, and motion of the liquid photoresists. In summary, our closed-loop TPL system can effectively compensate for two classes of errors: (1) errors generated at a low-frequency range (e.g., laser power drift, ambient temperature changes etc.); and (2) errors that cause part height variation (e.g., over/under laser exposure). More discussions on the fabrication error sources are in the Supplementary Information.

### Fabrication of large-scale optical devices via closed-loop two-photon lithography

**Fig. 5:**
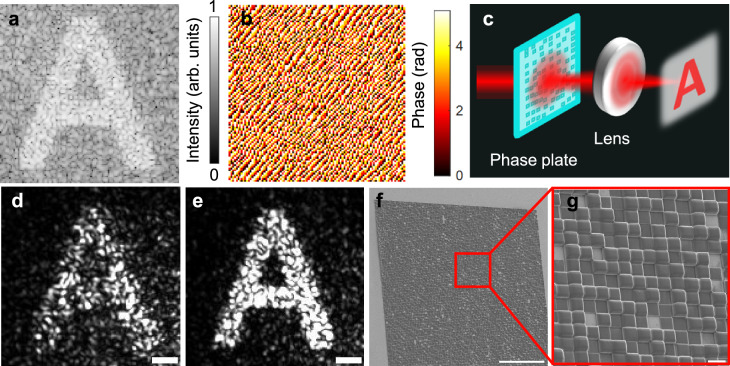
Design and characterization of a millimeter scale diffractive optical element, fabricated via open-loop and closed-loop two-photon lithography. **a**, **b** Simulated diffraction image and approximated 11-level phase profile of a 100 × 100-pixel diffractive optical element.**c** Setup to characterize the diffractive optical elements with an imaging lens and CCD camera. **d**, **e** Images recorded by the CCD camera for diffractive optical elements fabricated by open-loop and closed-loop two-photon lithography, respectively. Scale bars: 100 µm. **f** Scanning electron microscopy image of the diffractive optical element (with a 30-degree tilt angle), fabricated via closed-loop two-photon lithography. **g** Zoom-in view of the red box in (**f**). Scale bars are 200 µm for (**f**), and 10 µm for (**g**).

To demonstrate the efficacy of the closed-loop TPL system, we designed and fabricated a (1) large-scale DOE and (2) FZP lens. First, we designed an 11-level DOE with 100 × 100 pixels (pixel size: 7.90 µm × 7.90 µm) to shape a laser beam into a pattern of letter “A”. The corresponding phase profile is calculated by the iterative Gerchberg-Saxton algorithm^20^. Figure 5a, b present the simulated diffraction intensity and the approximated 11-level phase profile for the DOE, respectively. For comparison, the designed phase profile was printed via both open-loop and closed-loop TPL using the same printing parameters as Fig. 3. The printed structures by both methods underwent identical post-processing steps. To test the fabricated DOEs, a laser beam with a center wavelength of 800 nm was applied to illuminate the DOEs, which were positioned at the front focal plane of an imaging lens; to record the results, a CCD camera (mer-050-560u3m, Daheng Imaging) was placed at the back focal plane of the lens, as illustrated in Fig. 5c.

Figure 5d, e present the recorded diffraction images of DOEs fabricated by the open-loop and closed-loop TPL, respectively, where one may observe that the closed-loop result precisely resembles the designed diffraction image in Fig. 5a. To quantitatively compare the results, we calculated the signal-to-noise ratio (SNR) of the images (i.e., mean gray-level value divided by the standard deviation of background signals), for the closed-loop and open-loop DOEs are 13.93 and 4.94, respectively. This clearly shows that the closed-loop result substantially improved the SNR with superior contrast. Figure 5f and g present the SEM image of the post-processed DOE fabricated by closed-loop TPL, confirming the quality of the close-loop fabrication results. (See Supplementary Fig. 2 for more details).

**Fig. 6:**
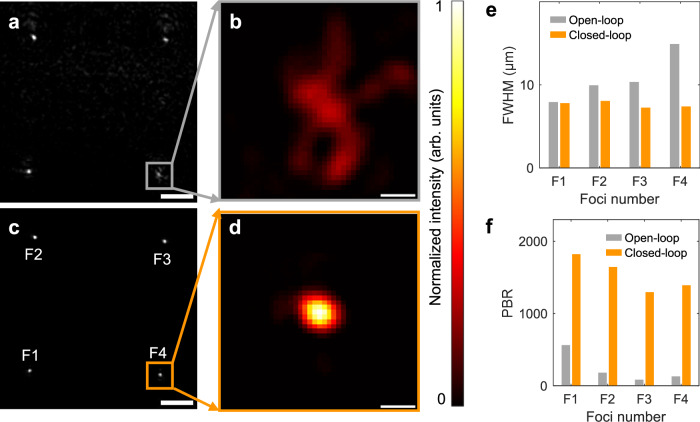
Imaging results of Fresnel zone plate lenses fabricated by open-loop and closed-loop two-photon lithography. **a, c** Measured four focal spots of the Fresnel zone plate lens, fabricated by open-loop and closed-loop two-photon lithography, respectively. **b, d** Zoom-in views of the boxes in (**a**) and (**c**), respectively. Scale bars are 100 µm for (**a, c**), and 10 µm for (**b, d**). **e** Measured the full width at half maximum (FWHM) of the four foci in (**a, c**). **f** Measured the peak-to-background ratio (PBR) of four foci in (**a, c**).

Next, we designed and fabricated an eight-level phase-type FZP lens^21,22^ via both open-loop and closed-loop TPL. The FZP lens was designed for 800-nm light source with four foci, each with a focal length of 4 mm; the FZP lens has a size of 800 × 800 µm^2^. (The phase profile of the FZP lens is presented in the Supplementary Fig. 3). Figure 6a–d present the characterization results of the FZP lenses. (See Supplementary Methods and Supplementary Fig. 5 for the FZP characterization setup). Specifically, Fig. 6a, c present the recorded four focal spots of the FZP lenses fabricated by the open-loop and closed-loop TPL, respectively. Figure 6b, d present the zoom-in views of the fourth focal point (i.e., F4) in Fig. 6a, c respectively with normalized 1D intensity profiles (across the center of the beam), where one may observe that the results in Fig. 6d shows superior focus quality with a full width at half maximum (FWHM) of 7.4 μm, which matches well with the designed value (i.e., 7.392 μm without noises; see details in Supplementary Fig. 6). Figure 6e presents the measured FWHM for all four foci of the FZP lenses fabricated via open-loop and closed-loop TPL. For the open-loop FZP lens, the measured FWHM is 7.93, 9.95, 10.35, 14.92 µm (See Supplementary Fig. 4 for details.) with a uniformity of 67.41% (Supplementary Methods). In contrast, the closed-loop FZP lens has a measured FWHM of 7.80, 8.06, 7.26, 7.39 µm with a uniformity of 94.74%, which outperforms the open-loop method by 27.33%. This result verified that the closed-loop TPL system can fabricate high-quality large-scale FZP lenses with substantially improved reproducibility. Lastly, Fig. 6f presents the peak-to-background ratio (PBR) (i.e., ratio of the mean focus intensity to the mean intensity outside the focus^23^) of each focus from both FZP lenses, where the open-loop and closed-loop PBR (from F1 to F4) are calculated to be (564, 181, 85, and 130) and (1820, 1643, 1296, and 1392), respectively. From the results, one may find that the closed-loop FZP lens shows a one order of magnitude higher PBR. From the results, we may conclude that the significant deterioration in the FZP lens performance (fabricated via open-loop TPL) is mainly due to systematic height errors that accumulate over time. The observed decrease in PBR is also in good agreement with the simulation results showing that height errors will reduce the PBR for FZP lenses (Supplementary Fig. 6). The high PBR values and uniform FWHM verify that the closed-loop TPL has achieved real-time compensation for fabrication errors, which will improve the system reproducibility over long printing sessions.

## Discussion

Comparing with previously reported TPL methods, our TPL platform achieved precise height control (i.e., within 100 nanometers) over long two-photon printing sessions ( > 10 hours), which is enabled by the integrated dual-comb module and real-time closed-loop control. Although the closed-loop method in principle can be simultaneously applied to control the lateral dimension of a designed part, we did not demonstrate that, as the lateral imaging resolution of the dual-comb system is less than the resolution of TPL. Note that the in-plane resolution may be improved by using a high NA objective lens (e.g., a 100× oil-immersion/NA 1.45 lens) at the expense of a smaller work field and shorter working distance. Also, under our controlled printing conditions (e.g., laser power and dose), the voxel aspect ratio can be maintained at ~1:2 as the laser dose slightly varies (Supplementary Fig. 8). This relation can be used to predict the lateral dimension of a thin structure based on high-precision height measurement. On the other hand, the precision of height detection and control may be further improved by selecting a more accurate fitting function or by increasing the data sampling time, at the expense of temporal resolution and total fabrication time. Notably, improved hardware such as high-speed data acquisition cards and GPU may simultaneously achieve better precision and higher speed. Overall, the closed-loop platform presents an economic and highly flexible (i.e., operation in both transmissive and reflective modes for transparent and opaque substrates, respectively) TPL solution. With the substantially improved quality, stability, and reproducibility, the closed-loop TPL platform may find important applications in fabricating large-scale devices in different fields such as optical devices for 6 G communication, data storage, ultra-fast computing, and 3D display, etc.

## Methods

### Two-photon lithography fabrication protocol

First, a glass or silicon substrate is sonicated in acetone and isopropanol solutions sequentially, each for 10 min. The substrate is then dried at 100 °C for 1 min and cleaned via O_2_ plasma for 2 min. For printing, the cleaned substrate is placed on the sample stage; photoresists (IP-L 780 or IP-Dip, Nanoscribe GmbH), which serve as the immersion fluid of the objective lens, are dispensed on the substrate via a pipette. Next, a femtosecond laser beam is focused on the photoresist, and TPP occurs at the laser focus. After TPL, the patterned substrate is post-processed in propylene glycol monomethyl ether acetate for 15 min, followed by isopropanol for 10 min. Finally, the developed structure is dried in the open air at room temperature.

### Height measurement

A dual-comb heterodyne detection method is used to characterize phase changes of the structures (being printed) in photoresist, which serve as the input signals for the closed-loop TPL platform. The recorded interferograms are then passed through a low-pass filter to only keep interference signals lower than 40MHz. The temporal interference signals are expressed as

1 $$V\left({{{\rm{t}}}}\right)\propto {\sum}_{{{{\rm{m}}}}}\{\exp \left[j2{{{\rm{\pi }}}}\left({f}_{1{{{\rm{m}}}}}-{f}_{2{{{\rm{m}}}}}\right)t\right]\cdot {A}_{1m}{A}_{2m}\cdot exp \left[j\left({\varphi }_{1m}-{\varphi }_{2m}\right)\right]\}$$

where ${A}_{1m.}$ and ${A}_{2m}$ are the amplitudes of Comb1 and Comb2, respectively; ${f}_{1m}$ and ${f}_{2m}$ are the optical frequencies of Comb1 and Comb2; ${\varphi }_{1m}$ and ${\varphi }_{2m}$ are the constant phases of the two combs, carrying the phase information along the dispersed direction.

Next, the phase spectrum ${\varphi }_{1m}-{\varphi }_{2m}$ of the interference signal is extracted by performing a Fourier transform (Note that the time-dependent term (i.e., *t* = 0) in each interferogram is neglected.), and subsequently averaging the results in the spectral domain. We then subtract the phase spectrum after TPL from the spectrum before TPL at the same position^16^. The phase difference $\varDelta \varphi$ induced by RI change is given by

2 $$\varDelta \varphi={\varphi }_{2m}^{{\prime} {\prime} }-{\varphi }_{2m}^{{\prime} }$$

where ${\varphi }_{2m}^{{\prime} {\prime} }$ and ${\varphi }_{2m}^{{\prime} }$ are the phase spectrum of Comb2 after and before TPL, respectively. Assuming part shrinkage after post-processing is negligible, the phase difference within the photoresist in the transmission mode is given by

3 $$\varDelta \varphi=\frac{2\pi \varDelta n}{{\lambda }_{0}}\cdot h$$

where $\varDelta n$ is the RI difference induced by TPP (i.e., ~0.035 for IPL-780), ${\lambda }_{0}$ is the wavelength of dual-comb laser in air (i.e., 1052 nm), and *h* is the height of post-processed structure. Therefore, the phase difference is linearly related to the height of the cure structures. Note that the measured phase difference of a structure in the reflection mode is twice than that in the transmission mode, which is caused by the substrate reflection.

### Algorithms for closed-loop two-photon lithography control based on phase feedback

The control algorithm is designed to approximate the set phase gradually until the deviation from the target is less than the preset threshold. The control algorithm is programmed and implemented in the TPL system via Python. Since curing of photoresists is irreversible, the laser exposure process starts from a lower phase than the target value. In the experiments, the system starts by using half the predicted dose (i.e., number of laser pulses at a constant laser power); and the real-time measured phase differences update the dynamic model at every new focus position. After the first loop, we approximate the target gradually based on the first-order iterative algorithm:

4 $$N\left({\varphi }_{{{{\rm{t}}}}+1}\right)=N\left({\varphi }_{{{{\rm{t}}}}}\right)+r\cdot \nabla N\left({\varphi }_{{{{\rm{t}}}}}\right)\cdot \left({\varphi }_{{{{\rm{target}}}}}-{\varphi }_{{{{\rm{t}}}}}\right)$$

where $N\left({\varphi }_{t+1}\right)$ and $N\left({\varphi }_{t}\right)$ are the number of exposed laser pulses at time *t* + 1 and *t*, respectively. $\nabla N\left({\varphi }_{t}\right)$ is the gradient of the mathematical phase model at ${\varphi }_{t}$. Moreover, to control the approximating step, the approximating ratio $r$ is set to 0.5 or less, which is related to the accuracy of height control.

## Supplementary information

Supplementary Information

Transparent Peer Review file

## Data Availability

The data generated and analyzed in this study are included in the main manuscript and the Supplementary Information. Additional information is available from the corresponding author (S.C.C.) upon request.

## Code availability

The custom codes used in this study for each control algorithm and the DOE simulation code are available from the corresponding author upon request.
