# Supplementary material for: Closed-loop high-precision two-photon lithography based on a multiplexed single-cavity dual-comb laser
Source: Nat Commun. 2026 Jun 3;17:7135. doi: 10.1038/s41467-026-73972-7 (PMC13396189; doi:10.1038/s41467-026-73972-7)
Supplement: Supplementary file 1 — Supplementary Information [file 41467_2026_73972_MOESM1_ESM.pdf]

Supplementary Information

Closed-loop High-precision Two-photon Lithography based on a Multiplexed Single-cavity Dual-comb Laser

Yalan Yu[1,†], Zhiwei Zhu[1,†], Benjamin Willenberg[2], Justinas Pupeikis[2], Christopher R. Phillips[2], and Shih-Chi Chen[1,3,*]

Affiliations:

[1]Department of Mechanical and Automation Engineering, The Chinese University of Hong Kong, Shatin, N.T., Hong Kong.

[2]Department of Physics, Institute for Quantum Electronics, ETH Zurich, Auguste-Piccard-Hof 1, 8093 Zurich, Switzerland.

[3]Centre for Perceptual and Interactive Intelligence, Hong Kong Science Park, Shatin, N.T., Hong Kong.

[†]These authors contributed equally to this manuscript: Yalan Yu, Zhiwei Zhu.

*Corresponding author: S.C. Chen. (email: scchen@mae.cuhk.edu.hk)

This PDF file includes

Supplementary Methods

Supplementary Figures 1-7

Supplementary References

Supplementary Methods

System configuration

Supplementary Figure 1 presents the detailed optical setup of the closed-loop TPL system, which includes a raster-scanning TPL fabrication module and a dual-comb metrology module to measure the phase differences in situ during the printing process.

The laser beam used for TPL fabrication is from Comb1. First, Comb1 is pumped into an OPO cavity to generate a 1600-nm beam, and then frequency-doubled to 800 nm via second harmonic generation (SHG). The femtosecond laser for TPL has a center wavelength of 800 nm, average power of 290 mW, and pulse duration of 151 fs (from 1.8W input of Comb1). The 800-nm laser beam is relayed to an acousto-optic modulator (AOM, MLP210-1DC, Gooch & Housego) for exposure control and next a two-axis galvanometric scanner (Galvo, QS20XY-AG, Thorlabs, Inc.), which performs two-dimensional raster scanning (i.e., in-plane two-photon printing). Note that the frequency of the AOM and galvo mirrors in our experiments were set to 800 kHz and 2 kHz, respectively. The polarization of the laser beam can be adjusted by a half-wave plate ($\lambda/2$) to maximize the diffraction efficiency of the AOM. An oil-immersion objective lens (OL1, CFI S Plan Fluor ELWD 40X, NA 0.6, Nikon) is used to focus the laser beam into photoresists. The sample (i.e., photoresists on a substrate) is mounted on a precision XYZ stage (L-511 for the z-axis; and L-509 for the x- and y-axis, Physik Instrumente) for large-scale TPL.

For phase measurement, the two combs are generated by the custom-built dual-comb laser system (1052 nm, 140 fs, 2 W, 80 MHz), where the repetition difference is set to 356 Hz in all TPL experiments. The polarization of Comb2 is adjusted by a half-wave plate ($\lambda/2$) and a polarizing beam splitter (PBS1). To measure the phase change in the photoresist, an optical mirror (M) relays Comb2 to a transmission diffraction grating (G1, T-1000-1040s, LightSmyth), which disperses the laser spectrum along one spatial direction. The dispersed laser is expanded by a pair of lenses (L3 and L4), reflected by an optical mirror (M), passes through a dichroic mirror (DM1) and lastly a 90:10 beam splitter (BS1). Comb2 then enters OL1 to form line illumination on the sample. Notably, the TPL printing process always occurs on the focus line of Comb2, where phase difference is measured.

To adapt to different printing applications, our system enables both reflection and transmission phase measurement. For reflection measurement, Comb2 is reflected by the substrate surface, and the dispersed beam is compensated by the same grating (G1) along the returning path. A quarter-wave plate ($\lambda/4$) is included to maximize the laser power passing through the PBS1. A video charge-coupled device (CCD) camera (Camera, Chameleon3 CM3-U3-50S5M, Point Grey Research) is used to monitor the TPP printing process in the laser focus. A collimated light emitting diode (LED1) at 580 nm is coupled to OL1 through a beam splitter (BS2), serving as the light source for epi-illumination.

For transmission measurement, Comb2 passes through the sample and is then collected by a second objective (OL2, CFI S Plan Fluor ELWD 40X, NA 0.6, Nikon), reflected by a dichroic mirror (DM2), and then dispersion-compensated by a pair of lenses (L5 and L6) and a grating (G2) to

eliminate the dispersion introduced in the illumination path. A transmissive illumination, generated by a light emitting diode (LED2) at 580 nm, is coupled to OL2 through DM2.

Note that all the dispersion compensated optical signals are interfered with a reference signal (i.e., Comb1) via a 50:50 fiber coupler (OC1 and OC2). The intensity and polarization direction of Comb1 can be adjusted by a half-wave plates ($\lambda/2$) and a polarizing beam splitter (PBS2). All interfered signals are acquired with a balanced differential detector (BPD1 and BPD2, PDB415C-AC, Thorlabs, Inc.), filtered by a low-pass filter and lastly digitized by a data acquisition board at a sampling rate of 500 MS/s.

Metric of uniformity.

The metric of uniformity is given as follows [1]:

$$U = (1 - \frac{V_{max} - V_{min}}{V_{max} + V_{min}}) \times 100\% \,, \tag{S1}$$

where U is the uniformity of a physical quantity, V_{max} and V_{min} are the maximum and minimum values of a physical quantity, respectively.

Characterization of FZP lenses

To measure the focal intensity profiles of the fabricated FZP lenses, Supplementary Figure 5 presents the schematic diagram of the optical characterization system. The light source is a custom-built femtosecond dual-comb laser system with a pulse duration of 151 fs and a central wavelength of 800 nm. An iris filter crops the incident beam to be approximately equivalent to the FZP lens radius. After the FZP lens, a 4-*f* system (*f*1 = 35 mm, *f*2 = 125 mm) magnifies the focused beam of the FZP lens; images of focal spot arrays are captured by a CCD camera (mer-050-560u3m, Daheng Imaging). In the experiments, the FZP lens is mounted on a three-axis translation stage; by adjusting the lens, beam profile images at the focal plane can be obtained accurately. In addition, the power of the laser beam is sufficiently reduced by using an ND filter to the extent that the camera is not saturated.

Fabrication Error Analysis

We summarize representative error sources observed in the experiments (not an exhaustive list) that can be corrected by our method:

- Substrate warpage and surface unevenness, especially deformation at the hundred-nanometer scale and larger.
- Photoresist stability issues, including photoresist (fluid) motion on the substrate and volatility under ambient air conditions.

- Fluctuations/variations in ambient operating temperature and humidity.
- Laser output intensity instability.

It is important to note that a prerequisite of our closed-loop correction method is that repeatable and precise components (such as precision XYZ stages, galvo scanners, and a vibration isolation table) are used in the TPL system. (Note these components are already used in most TPL setups which can print fine structures (< 200 nm) through open-loop calibration.) In these open-loop systems although high precision can be achieved through calibration, the optimal condition often cannot be maintained for too long (e.g., a few hours) owing to parameter drift. It is under this typical condition, our closed-loop solution can quickly identify the optimal condition with simple calibration and maintain the optimal writing condition indefinitely until the end of the fabrication.

Supplementary Figures

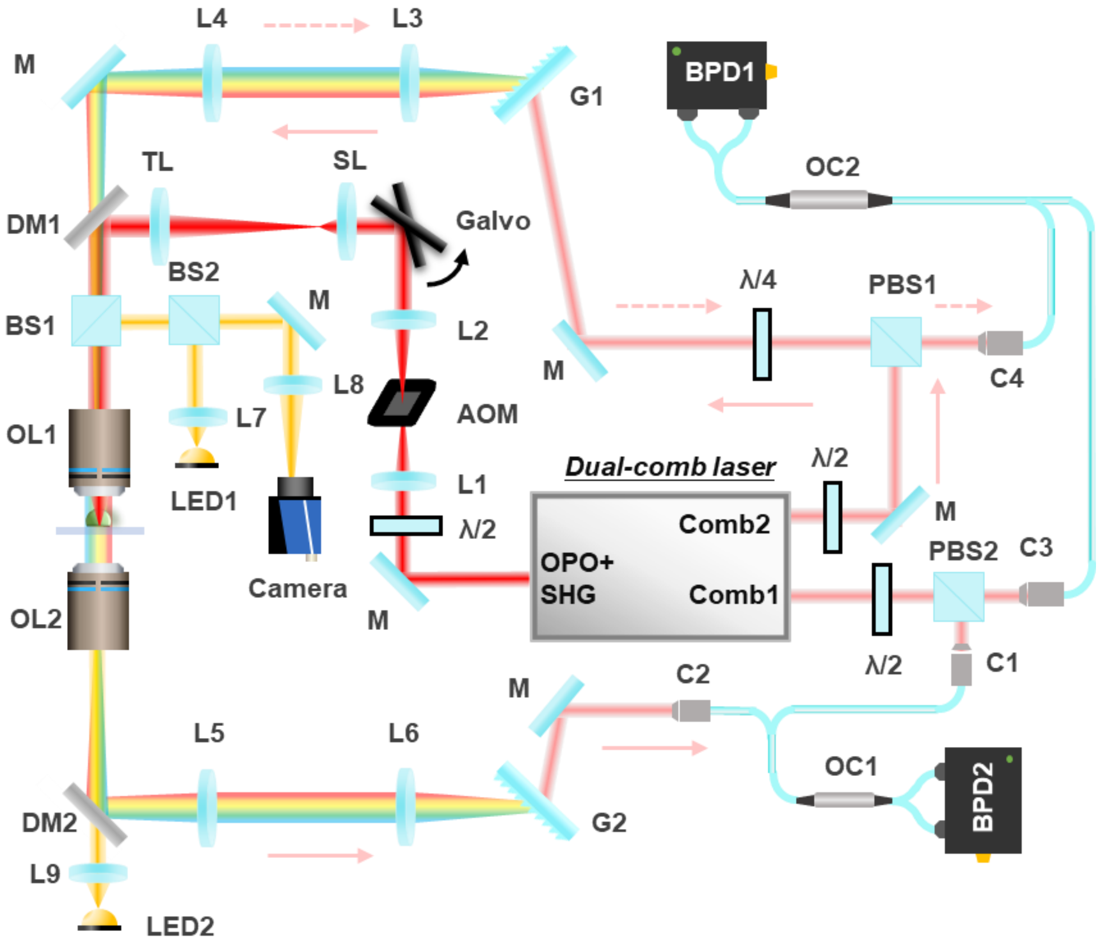

Supplementary Figure 1. Optical setup of the closed-loop TPL system. PBS: polarization beam splitter; BS: beam splitter; C: fiber collimator; OC: fiber optic coupler; $\lambda/4$: quarter-wave plate; $\lambda/2$: half-wave plate; M: mirrors; G: transmission diffraction grating; OL: objective; DM: dichroic mirror; AOM: acousto-optic modulator; Galvo: galvanometric scanner; SL: scan lens; TL: tube lens; BPD: balanced photodetector; LED: light emitting diodes; and L1-L9: lenses.

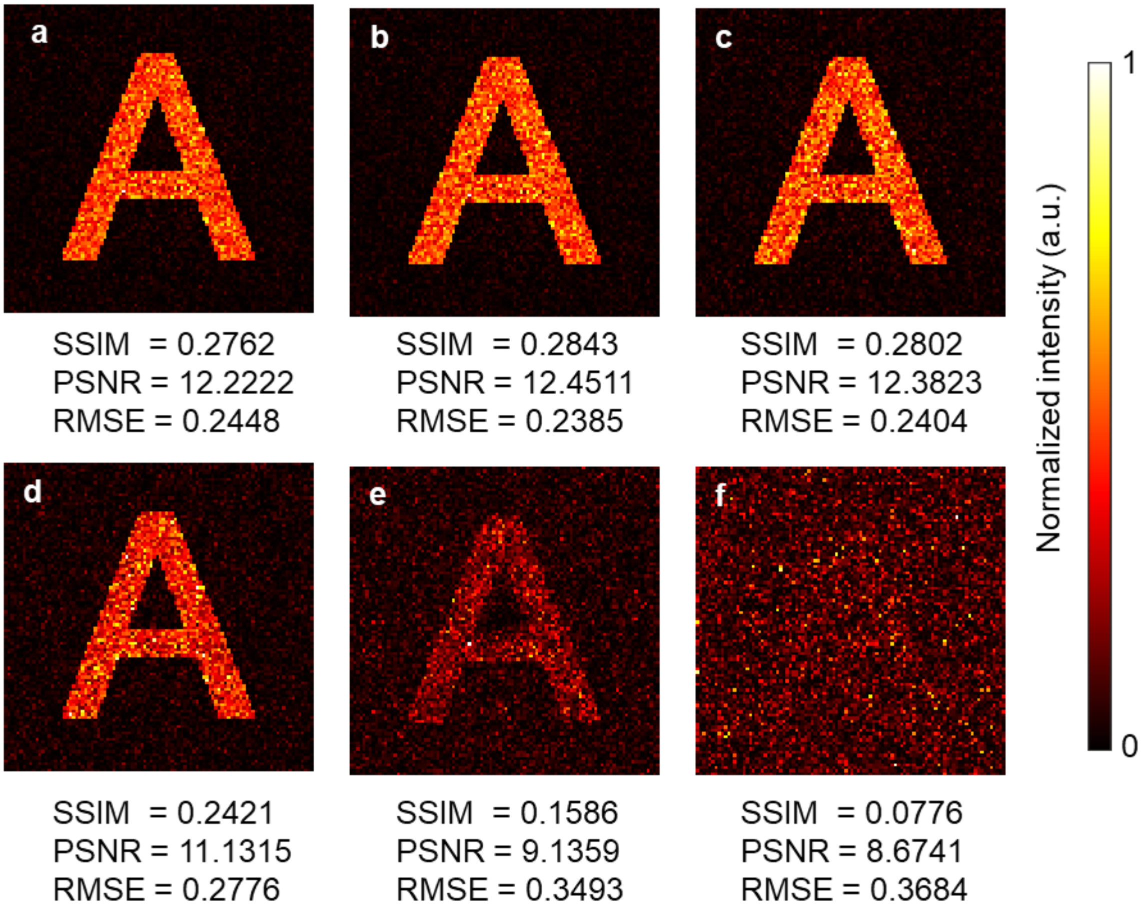

Supplementary Figure 2. Effect of fabrication errors (i.e., errors in pixel height) to the designed DOEs. Simulation results of reconstructed diffraction image from the fabricated structures with different height errors. (a)-(f) Phase errors in the photoresist were set to 0.0, 0.02, 0.05, 0.1, 0.2 and 0.3 rad, respectively.

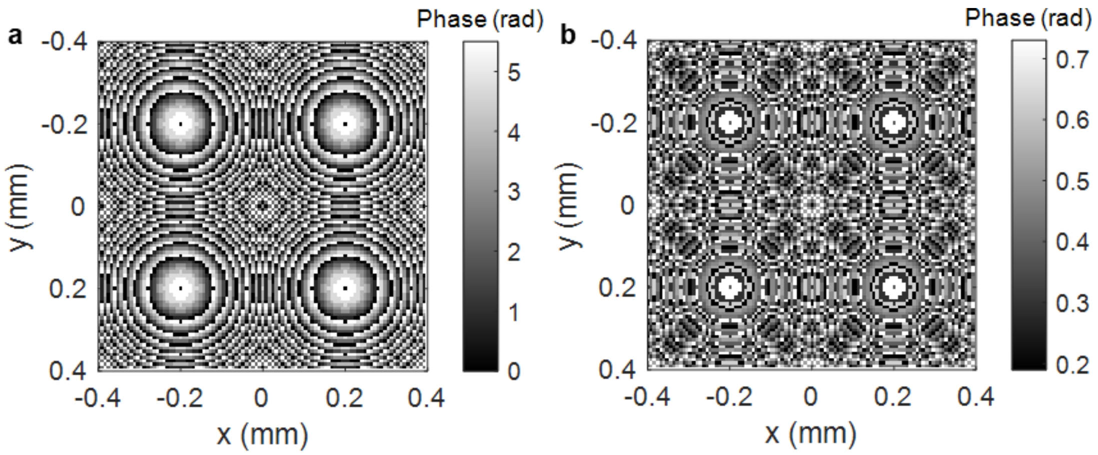

Supplementary Figure 3. Phase profile of the FZP lenses in air (a) and within photoresist (b).

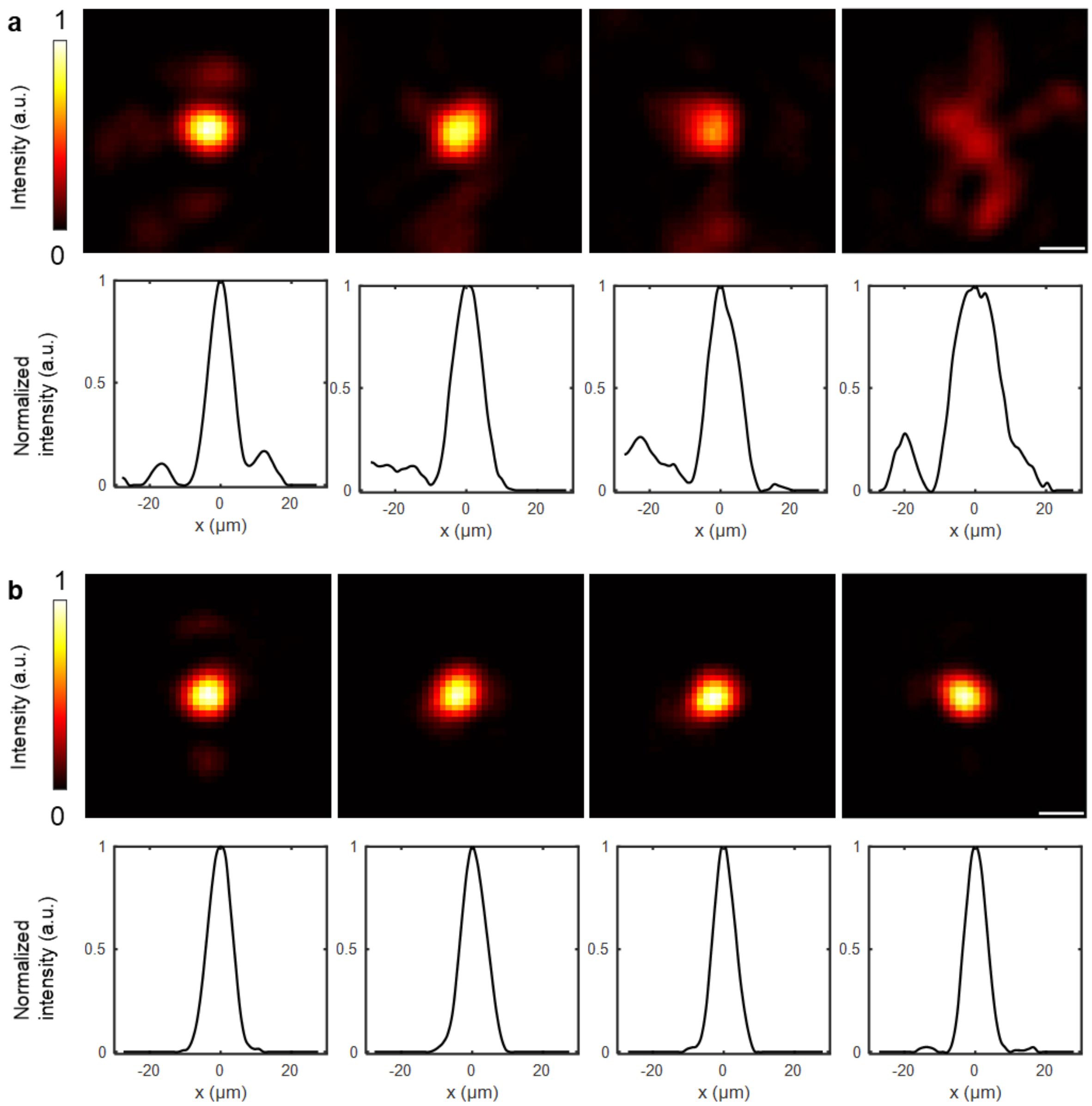

Supplementary Figure 4. Measured four focus intensity profiles of the Fresnel lenses fabricated by the (a) open-loop TPL system, and (b) closed-loop TPL system, corresponding to F1, F2, F3 and F4 (from left to right) in Figure 6, respectively. In (a) and (b), the top row presents zoomed-in intensity profiles, and the bottom row presents 1D normalized intensity distributions. Scale bars: 10 μm.

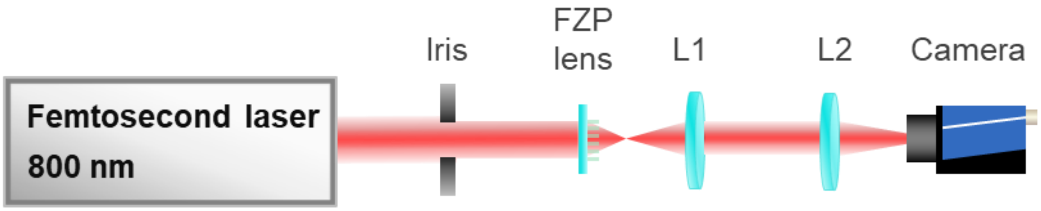

Supplementary Figure 5. Schematic diagram of the FZP lens characterization system. L1-L2, lenses (focus lengths: 35, 125 mm, respectively).

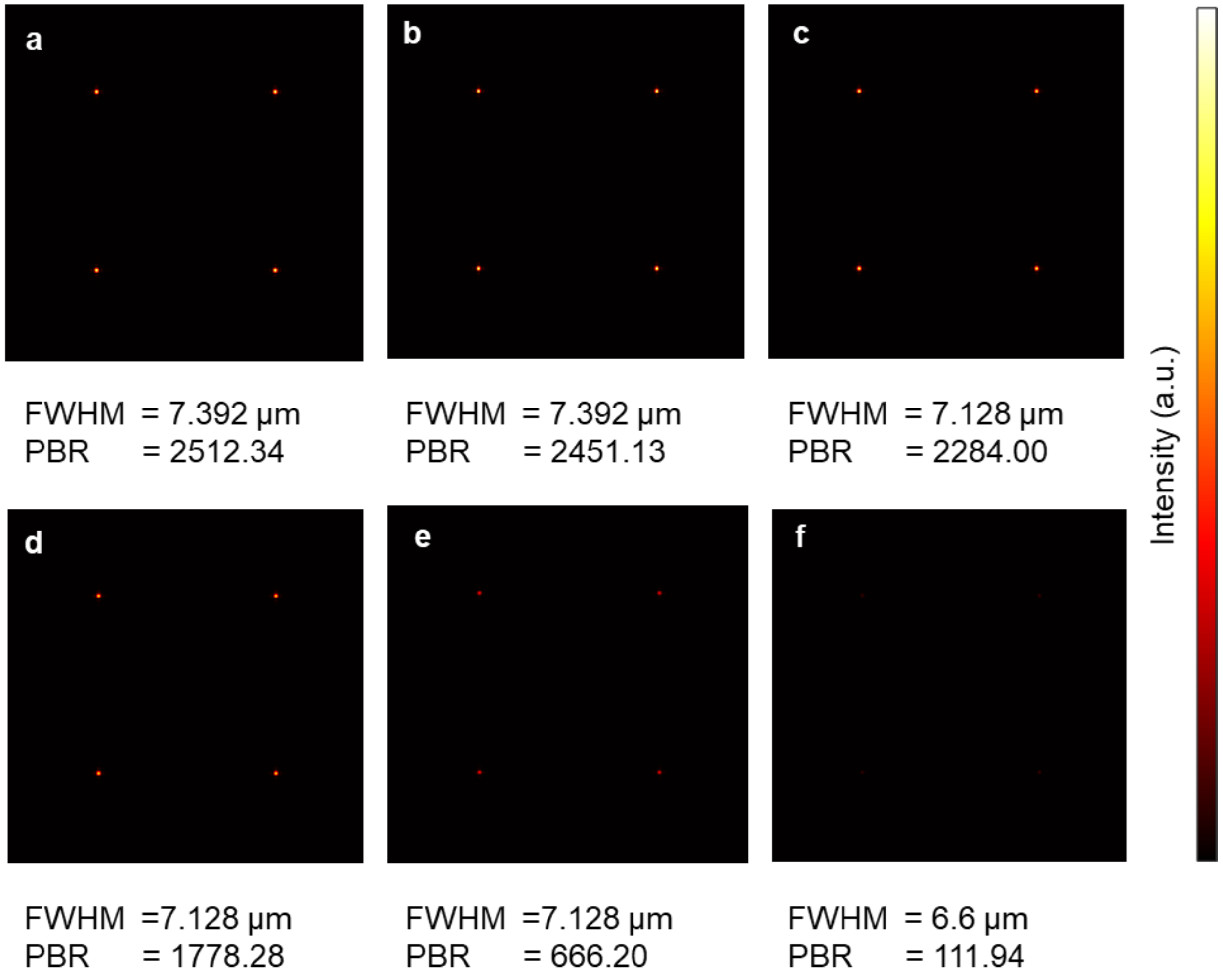

Supplementary Figure 6. Simulated focusing results of the FZP lenses (2 × 2 focal points) with different height errors. (a)-(f) The phase errors in the photoresist were set to 0.0, 0.02, 0.05, 0.1, 0.2 and 0.3 rad, respectively.

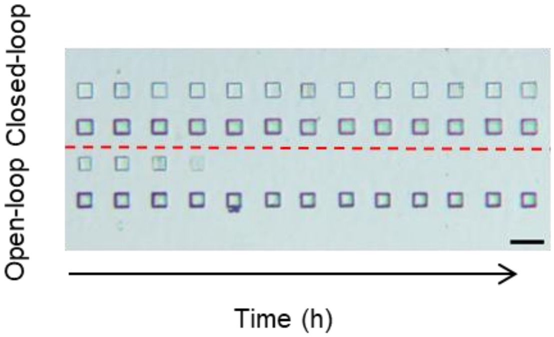

Supplementary Figure 7. Optical image of the post-processed cuboid structures in Figure 4c,d. The control setpoints for the first and third rows are 1.0 μm; and those for the second and fourth rows are 4.8 μm. Scale bar: 20 μm.

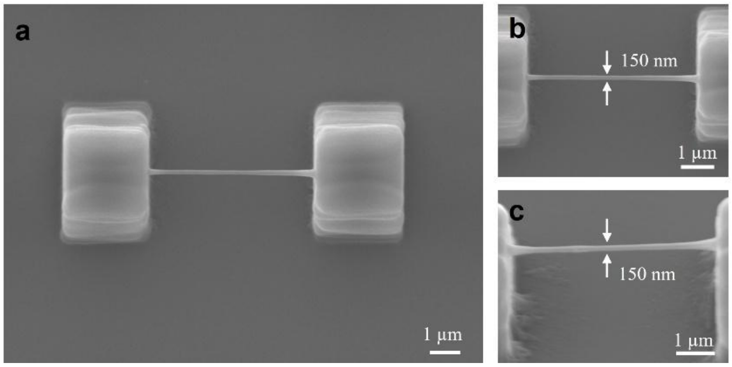

Supplementary Figure 8. Point-spread function (PSF) characterization via fabrication of a suspended-nanowire structure. (a) SEM image of the entire nanowire structure, supported by two bases; (b) suspended wire with 0° stage tilt (top view) that was measured to have a lateral dimension of 150 nm; and (c) suspended wire with 30° stage tilt that was measured to have a projected width of 150 nm, which corresponds to an axial dimension of 300 nm. The results in (a) and (b) indicate that the voxel aspect ratio is approximately 1:2 under our experimental conditions.

Supplementary References

[1] D. Chen, S. Gu, S.-C. Chen, *Optics and Lasers in Engineering* **2021**, *142*, 106604.
