# Supplementary material for: Closed-loop high-precision two-photon lithography based on a multiplexed single-cavity dual-comb laser
Source: Nat Commun. 2026 Jun 3;17:7135. doi: 10.1038/s41467-026-73972-7 (PMC13396189; doi:10.1038/s41467-026-73972-7)
Supplement: Supplementary file 2 — Transparent Peer Review file [file 41467_2026_73972_MOESM2_ESM.pdf]

nature portfolio

Peer Review File

Closed-loop High-precision Two-photon Lithography based on a Multiplexed Single-cavity Dual-comb Laser

Corresponding Author: Professor Shih-Chi Chen

This file contains all reviewer reports in order by version, followed by all author rebuttals in order by version.

Version 0:

Reviewer comments:

Reviewer #1

(Remarks to the Author)
Author report insitu monitoring of the TPL fabrication to improve the reproducibility. The reported experimental setup is capable of doing simultaneous fabrication, monitoring and error correction. Literature already demonstrates sub-100 nm feature fabrication with reliable reproducibility, particularly through DOE-based MPL approaches and highly sensitive photoresists. In this context, the authors' claim of achieving sub-200 nm resolution with a 10-hour fabrication time appears dated [1], [2]. The authors outline the in-situ monitoring strategy and compare phase imaging, optical coherence, and diffraction-based tomography techniques. However, they should also discuss the limitations of these previously reported methods. In particular, beam profile correction during live fabrication is crucial for reproducibility, yet this aspect is not addressed. Manuscript lacks technical clarity and finess and should not be considered for publication. Insitu monitoring has been an active area of research and TPL should also benefit from it. Combination of insitu monitoring with TPL should be shown with best linewidths which is around 100nm and with free standing structures not on cuboid where tiling error is difficult to identify. Few other details are missing which are mentioned below for the authors to improve manuscript in future.
Additionally, the claim that "a precise and robust closed-loop control method for TPL to improve its quality and reproducibility has yet to be developed" should be clarified. Are Chen et al. the first to assert this? If so, a stronger comparison with existing control and feedback strategies in TPL is needed to justify the novelty of the approach. The authors report using a custom single-cavity dual-comb laser (1052 nm, 140 fs, 2 W, 80 MHz) and perform TPL via wavelength conversion and SHG, yielding 290 mW average power and 151 fs pulses. Since synchronized two-axis galvanometer scanners are employed, the manuscript should specify the XYZ stage model, resolution, and travel range. These details are necessary to assess the fabrication accuracy and validate the claimed performance.
Is the grating G2 same as G1? if not mention the details of the G2 as well. Which process parameters are actually included in the closed-loop control? For example, does it track laser power, writing speed, exposure dose, focal position, and objective selection? Also, would system calibration need to be redefined if the objective lens is changed, e.g., from a 40×/NA 0.6 to a 100× oil-immersion/NA 1.45 lens?
The authors report an open-loop error of 1139 nm. This appears quite large. What factors contribute to such a high value, and is this measurement obtained from their own system or sourced from previous literature? The reported structures show curved edges rather than the sharp boundaries expected for a cuboid. The authors should clarify why the edges are rounded and whether this results from voxel shape, beam aberrations, or processing parameters.
Additionally, if the writing speed is 100 mm/s for structures of 8×8×1 µm³ and 8×8×4.8 µm³ with a 100 nm hatch spacing, it is unclear why the total fabrication time is 12 hours. The slicing distance is also not specified, which is necessary to understand the total voxel count and actual writing duration.
The reported average height errors for the 1.0-µm and 4.8-µm cuboids are 0.130 µm (s.d. 0.088 µm) and 0.084 µm (s.d. 0.069 µm), respectively. While the authors attribute these deviations to temperature fluctuations and mechanical vibrations, such factors can generally be minimized under controlled lab conditions using stable temperature/humidity regulation and pneumatic isolation tables. Laser power drift typically affects much larger structures, so its role in height variation at these small scales should be reconsidered or clarified. Clarity on how the closed-loop system actively drives the process toward the target fabrication values is insufficient in the current text and needs further explanation. How do the authors plan to control or compensate for beam distortions in real time?
Typographical errors:
Line 84: 40X should be mentioned as 40×
Figure caption 3: inconsistent usage of (), please follow the same usage throughout the aricle.
Figure caption 3: SEM image (a), AFM image (b),….. can be rewritten as: a. SEM image, b. AFM image…

[1] Liu, Tianqi, Peipei Tao, Xiaolin Wang, Hongqing Wang, Minfei He, Qianqian Wang, Hao Cui et al. "Ultrahigh-printing-speed photoresists for additive manufacturing." Nature Nanotechnology 19, no. 1 (2024): 51-57.
[2] Jiao, Binzhang, Fayu Chen, Yuncheng Liu, Xuhao Fan, Shaoqun Zeng, Qi Dong, Leimin Deng, Hui Gao, and Wei Xiong. "Acousto-optic scanning spatial-switching multiphoton lithography." International Journal of Extreme Manufacturing 5, no. 3 (2023): 035008.

Reviewer #2

(Remarks to the Author)
The authors present a study on the development of a two-photon lithography technique integrating a smartly developed and integrated closed loop to measure in real time variations of the actual sample modified, providing high control over the final height resolution imprinted. The approach enables measuring a real-time phase and height maps that feed a closed-loop controller that iteratively adjust the printing parameters regarding with the laser pulse energy/dosage, scanning rate and exposure time. The error using the close loop system is always smaller over long processing times compared to that of operating the same system under an open loop configuration. These results apply to the specific case of the fabrication of a diffractive optical mask. The approach presents high height reproducibility and stability. I consider the paper can be accepted for publication in Nature Communications only after some minor clarifications from the authors regarding:
- Specific calibration requirements for different polymer resins reduce compatibility with other systems. It is already highlighted that these results apply only to the photoresist IP-L 780. Additional comments should be added pointing to the parameters that need to be considered towards applicability of the current approach with other photoresists.
- As lateral resolution is not increased, but on the contrary, it is worsened, the applications for which the current approach is limited to flat phase masks and plates to be later used as diffractive optical elements. As resolution increase from the optical point of view are feasible, the authors should comment on possible approaches that would increase lateral resolution.
- Data included in Figure 4c suggests that after 4 hrs, the open-loop configuration would not work anymore. It is not clearly stated why. Can the authors comment on that?
- From the experimental point of view, I wonder if the lack in lateral resolution comes from the fact that the beam is being focused by a regular lens and not by an F-theta lens. The beam is always entering at different positions because of the galvanometric scanner is scanning the beam before it enters the objective. Therefore, it will already produce a focal spot at different planes (or following a spherical surface). Can the authors include estimated variation given by this effect? On that line, would a high numerical aperture objective improve lateral resolution? The main challenges for using different optics (high NA for example) should be mentioned.
- The nominal pulse duration in this case is in the hundreds of fs. There are many optical elements that may induce temporal dispersion on the femtosecond pulses, and when the pulses arrive at the irradiation region, they are surely longer in time. Is the pulse duration and the pulse dispersion in time a parameter that affects considerably the printing outcomes? Resin compatibility may be affected radically when the pulse is a lot longer than nominal values at the exit of the laser source. Additional comments should be added in that regard.
- Final comment is regarding the experimental details on the characterization of the Fresnel Zone Plate lenses. Although the experimental setup is included in the supplementary material, I find it lacks description of elements such as the incorporation of quarter wave plates in the characterization path, and the use of an additional balanced photodiode. This part is just for the characterization, but for the sake of completeness, additional comments should be included.

Reviewer #3

(Remarks to the Author)
This article presents a novel phase imaging technique to measure the change in phase that occurs during two-photon lithography (TPL) and applies it to demonstrate higher-precision printing of optical elements, such as diffractive optical elements (DOE) and Fresnel zone plates (FZP). The improvement in precision was achieved through real-time feedback control, which has been challenging to achieve in TPL. Amongst the different types of functional materials that are produced by TPL (such as bio-scaffolds, mechanical metamaterials, etc.), optical elements tend to have more stringent precision requirements. Therefore, this work is timely and valuable for the practical implementation of TPL for industrial scale use. However, there are several issues with this study that require revision.

Major issues:
1. A clearer explanation of the premise of this study should be provided. In its current form, this article may be confusing to readers who are new to TPL. In general, voxel formation in TPL is remarkably repeatable. If the input parameters (such as laser power and scanning speed) are held constant, one expects to obtain repeatable voxel geometries. Lack of precision in large structures (i.e., assembly of voxels) usually arises from the inability to hold the process parameters constant (over a long period of time) or due to motion errors (i.e., errors in alignment, positioning, or speed).

1.1. The authors have not specified the sources of error that are being compensated here. Are they compensating for motion errors (such as XYZ stage drifting away from its set point height, a pre-existing tilt of the substrate, or some other error motion) or errors due to variation in the laser power over time? Can the authors specify the error sources being compensated here and support their claims with relevant data, such as laser power drift over time, position of the Z stage over time, etc.?

1.2. From Figure 4c, it appears that the error being compensated is the tilt of the substrate. If so, would it not be easier to measure the tilt of the stage before printing and then adding this as a compensation term to the commanded Z location of the stage? Can the authors compare the tradeoffs of their approach and the tilt compensation approach?

1.3. The compensation approach seems to rely on increasing the amount of printing (by increasing the number of pulses) so that more printing can occur in regions where the focal plane has moved away from the substrate (and thus has a lower printed height). Would this approach work well in other scenarios, such as those listed in the introduction (vibration, and laser power fluctuations), particularly with respect to the time scales of these other error sources.

1.4. Related to the point above, increasing the amount of printing will not only increase the height of the structures but also the width of the structures (and thereby introduce errors). What is the typical deviation of the measured width from the designed width of the blocks (during close-loop operation)? The error introduced along the lateral direction may not be an issue for DOE and FZP structures, as the optical performance is more sensitive to height errors. However, for other applications, this may become a limitation. Can the authors comment on the generalizability of their compensation approach in light of this limitation?

1.5. The authors have not provided any comparison of the quality of their optical structures (DOE or FZP) with respect to those printed by others (for example Reference 21). Did past researchers experience same errors and how did they solve it? Now that the authors have a new control strategy, what improvements can the readers expect over the state-of-art of DOE and FZP fabrication?

2. A major assumption in this work is that the change in refractive index (RI) of the resist due to curing is constant (Equation 3). However, it is well-known that the change in RI depends on the dosage during TPL printing (for example, see: https://doi.org/10.1364/OL.44.000029). For large dosages, such as in the lower-right quadrant of Figure 2(d), the authors' approximation may be reasonable. However, is Equation 3 still valid at low dosages, such as in the upper-left quadrant of Figure 2(d)? For example, could the authors provide a comparison of measured phase versus measured height (such as Figure 2(e)) for low dosage conditions? Is the trend still linear? Related to this, what was the power for the data shown in Figure 2(e)?

3. The authors have used the number of pulses as the controllable parameter in this study, but it has not been defined. If the beam is not scanned, the interpretation is straightforward (as it is determined by the duration of exposure at any given location). However, what is the interpretation of this parameter when the beam is being scanned? Is it the number of pulses that are incident on any given material point (in which case it cannot be tuned without changing the scan speed) or is it the total number of pulses incident over a scanned area? If it is the later, it suggests that the same region is being scanned multiple times and changing the duration of exposure implies changing the number of times the same region is being scanned. If so, this should be specified in the text.

4. Can the authors clarify how the scanning of the imaging laser occurs? As the imaging laser forms a line illumination (and sensing occurs via a photodetector), presumably the laser should be scanned to generate 2D images such as those shown in Figure 3(c). However, it is not passing through the Galvo (laser through L4-M4-DM). How is the scanning achieved?

5. Can the authors clarify how the scanning of the printing laser (focal point) and the imaging laser (focal line) are synchronized in space and time and its impact on averaging of phase information? Depending on the scanning paths and the scan rates, the phase information captured by the detector can come from various different locations on the sample and from various instants of time. For the structures demonstrated here, this may not be an issue because the target geometry looks uniform over each 8 μm×8 μm slab. However, for more complex 3D geometries, this may become a limitation for real-time control. Can the authors comment on this?

6. What evidence can the authors provide in support of their claim in the Discussion section (lines 267-268) that their technique can be applied to control the lateral dimension of a designed part? As the phase measurement (Equation 3) is independent of the lateral geometry, it is difficult to appreciate how this technique can be applied to control lateral dimensions.

Minor issues:

7. In the abstract, the authors claim that laser power, scanning rate, and exposure time are being modulated. However, data for power and scanning rate modulation has not been presented. The authors may consider revising the abstract so that it is consistent with their work.

8. In the methods section (lines 296 -297), it is difficult to understand how the spectrum before TPL was obtained. This is because the optical elements for this measurement have not been shown in the main Figure 1 (but are shown later in the supplementary Figure 1). The authors may consider highlighting in the methods section that additional details on the optical setup are shown in the supplementary Figure 1.

9. Figures 6 (b) and (d) seem to have incorrect scale bars. The labeled lengths are inconsistent with the scale bar.

10. In the Introduction section (lines 36-37), the authors mention that in-situ imaging techniques are limited by diffraction. The authors may consider clarifying that the resolution of their technique is also limited by diffraction along the lateral dimension,

but that it is not diffraction-limited along the depth direction due to its interferometric phase measurement mechanism.

11. Consider using different marker styles for open versus closed loop in Figures 4(c) and (d).

Version 1:

Reviewer comments:

Reviewer #2

(Remarks to the Author)
The authors have incorporated and answered all the concerns, major and minor modificaitons proposed during the first round of reviews.
It has to be noted that additional data is now included into the new version of the paper, and that experimental details that previously were not reported are now included in full. In addition, new descriptions and figures were included, and additional discussion was added.
I condisider the paper can be now accepted for publication in Nature Communications, and congratulate the authors for the nice work.

Reviewer #3

(Remarks to the Author)
For the most part, the authors have provided satisfactory responses. However, the authors' unwillingness to appropriately revise their manuscript to clarify the scope of their approach is a cause for concern.

1. The authors continue to claim in their response that "we'd like to point out that identifying and eliminating the error sources is not the focus of this work because once closed-loop control is implemented, all errors from different sources will be effectively compensated." Claiming that all errors will be compensated is a very broad claim and it is not accurate. If the stages drift, their technique will not compensate for this error. For example, consider the target structure being a woodpile porous 3D structure with voxel-level nanowires (i.e., ~300 nm wide wires with 1000 nm pitch). If the non-repeatability in such a structure is due to drift in Z-motion stage or vibration in the galvo, their compensation technique will not be effective. The authors explain in their response that they believe that the errors that they are compensating arise due to changes in the response of the resist over time or due to non-flatness of the substrate. The authors should consider explicitly mentioning these potential sources in their manuscript (either in the main text or the supplementary material). Based on this, they should also consider grouping potential error sources into those that can be compensated by their technique and those that cannot be readily compensated by their technique. Being forthright about the capabilities and limitations of their work will not diminish the value of their work.

2. In their revision, the authors claim that the voxel aspect ratio is fixed at 1:2. This is not universally true in TPL as the aspect ratio can vary over a large range such as 1:1 to 1:6. The authors should provide evidence to support this claim or consider revising this argument based on the state-of-art of TPL.

Version 2:

Reviewer comments:

Reviewer #3

(Remarks to the Author)
The authors have satisfactorily revised the manuscript, and it is ready for publication.

Open Access This Peer Review File is licensed under a Creative Commons Attribution 4.0 International License, which permits use, sharing, adaptation, distribution and reproduction in any medium or format, as long as you give appropriate credit to the original author(s) and the source, provide a link to the Creative Commons license, and indicate if changes were made.
In cases where reviewers are anonymous, credit should be given to 'Anonymous Referee' and the source.
The images or other third party material in this Peer Review File are included in the article's Creative Commons license, unless indicated otherwise in a credit line to the material. If material is not included in the article's Creative Commons license and your intended use is not permitted by statutory regulation or exceeds the permitted use, you will need to obtain permission directly from the copyright holder.
To view a copy of this license, visit https://creativecommons.org/licenses/by/4.0/

Point-by-point response to reviewers' comments:

Reviewer #1 (Remarks to the Author):

Comment 1-1:

Author report in situ monitoring of the TPL fabrication to improve the reproducibility. The reported experimental setup is capable of doing simultaneous fabrication, monitoring and error correction. Literature already demonstrates sub-100 nm feature fabrication with reliable reproducibility, particularly through DOE-based MPL approaches and highly sensitive photoresists. In this context, the authors' claim of achieving sub-200 nm resolution with a 10-hour fabrication time appears dated [1], [2]. The authors outline the in-situ monitoring strategy and compare phase imaging, optical coherence, and diffraction-based tomography techniques. However, they should also discuss the limitations of these previously reported methods. In particular, beam profile correction during live fabrication is crucial for reproducibility, yet this aspect is not addressed. Manuscript lacks technical clarity and finess and should not be considered for publication. In situ monitoring has been an active area of research and TPL should also benefit from it. Combination of in situ monitoring with TPL should be shown with best linewidths which is around 100 nm and with free standing structures not on cuboid where tiling error is difficult to identify. Few other details are missing which are mentioned below for the authors to improve manuscript in future.

Response 1-1: Thank you for your comment. **However, we cannot agree with your assessment on our work. In particular, your statement that "literature already demonstrates sub-100 nm feature fabrication with reliable reproducibility" is incorrect.** Indeed, many prior works demonstrated 100-nm level feature sizes via TPL (In fact, some of the leading records were set by our team, e.g., S.K. Saha et al., *Science*, 366(6461): 105-109, 2019; and Ouyang et al. *Nature Communications*, 14: 1716, 2023.) However, these results were all achieved based on well calibrated laser system, exposure doses and strictly controlled environment; and more importantly such writing condition can easily drift away over time (>1-2 hours). **A turn-key TPL solution with reliable long-printing session (>10 hours) with ± 15 nm-level reproducibility has never been demonstrated before this work.** It is also worth to note that long printing time is a key requirement for TPL to fabricate large parts. The revised manuscript has provided all system and optical design details as well as closed-loop control details for expert readers to repeat our experiments and results.

It is true our method only provides suitable metrology precision for TPL in the axial direction (5-nm level precision via dual comb interferometry (DCI); diffraction limited in the lateral direction. See Reviewer 3's Response 3-1-4 for more details on predicting the lateral dimension.); and thus, our target application is to write flat structures such as diffractive optical elements, Fresnel lenses or complex phase plates as demonstrated in the paper. Although limited, our results have demonstrated that TPL can be a solution with comparable precision/repeatability with electron-beam lithography with 2-3 orders of magnitude fabrication rate, which is not only a major breakthrough in the field, but also a paradigm shift to the nanofabrication community and industry.

Regarding your comments on other in situ monitoring methods, we agree there have been quite some works demonstrating in situ monitoring of TPL processes (including our first demonstration in 2022: He et al., *Additive Manufacturing*, 60:103293, 2022.). **Yet, none of these methods were able to use the in situ measured data to close the loop of TPL to improve the TPL reproducibility over long printing time.** If the loop was not closed, the in situ monitoring optical metrology system is just a low-resolution alternative to SEM or AFM. The main challenges of closing the loop (i.e., using the measured data to control printing parameters in real time) include (1) to achieve meaningful precision for TPL optically (i.e., 10s of nanometer level); and (2) to rapidly process the measured data (e.g., phase changes or refractive index changes) to generate control signals and tune the printing parameters. **Our work presents the first demonstration of closed-loop TPL (via DCI) to fundamentally address the challenge of reproducibility.**

Regarding your comments on beam profile correction, beam profile is indeed one of the many parameters (including drifting laser power, temperature, humidity, mechanical vibration, and motions of the liquid resins etc.) that can influence the TPL writing results. However, our method can continuously monitor and control the part height at 10-nm level precision; thus errors induced by various factors are effectively compensated including beam profile change. With the DCI module, we no longer need to warm up lasers, nor perform any calibration experiments to achieve consistent 100-nm TPL precision, realizing turn-key TPL fabrication at 100-nm level precision.

Comment 1-2:

Additionally, the claim that "a precise and robust closed-loop control method for TPL to improve its quality and reproducibility has yet to be developed" should be clarified. Are Chen et al. the first to assert this? If so, a stronger comparison with existing control and feedback strategies in TPL is needed to justify the novelty of the approach.

Response 1-2: Thank you for your question. As explained in Response 1-1, our work is the first demonstration of closed-loop TPL to greatly improve printing reproducibility. Although there are different in situ monitoring methods reported previously, the measured data were not used to control the printing condition directly; and there were no existing control and feedback strategy in TPL to compare to. Note that resolution and repeatability (or reproducibility) are different concepts; TPL has 100-nm level printing resolution, but the repeatability is on the 100s nm - micron level for long-term printing without closed-loop control as can be seen in our experiments.

For your reference, Table R1-1 briefly compares our dual-comb method with other reported in situ metrology methods for TPL.

Table R1-1 Comparison of in-situ phase measurement techniques in TPL

Techniques	Lateral resolution	Height sensitivity	Speed	Measurement mode	References
OCT	1.9 μm	2.7 μm	30 s per circle	Only reflective	[R1]
ODT	diffraction-limited	diffraction-limited	acquisition time of 6 ms	Only transmissive	[R2]
QPI	--	--	>10 min	Only transmissive	[R3]
Our method	1.1 μm	**4.8 nm**	**2.1 s per circle**	**Both reflective and transmissive**	

Comment 1-3:

The authors report using a custom single-cavity dual-comb laser (1052 nm, 140 fs, 2 W, 80 MHz) and perform TPL via wavelength conversion and SHG, yielding 290 mW average power and 151 fs pulses. Since synchronized two-axis galvanometer scanners are employed, the manuscript should specify the XYZ stage model, resolution, and travel range. These details are necessary to assess the fabrication accuracy and validate the claimed performance. Is the grating G2 same as G1? if not mention the details of the G2 as well. Which process parameters are actually included in the closed-loop control? For example, does it track laser power, writing speed, exposure dose, focal position, and objective selection? Also, would system calibration need to be redefined if the objective lens is changed, e.g., from a 40×/NA 0.6 to a 100× oil-immersion/NA 1.45 lens?

Response 1-3: Thank you for your question. Below are more details about our experiments, which we have now included in the revised manuscript:

Regarding parameters of closed-loop control, we used an acousto-optic modulator (AOM) to control the number of laser pulses to achieve closed-loop control. The dose prediction function satisfies a precise convex and monotonically increasing relationship with the height of the printed structures, as shown in Fig. 2d and Fig. 4b.

Regarding system design and calibration, if changing the objective to a high-NA lens, the system needs to be calibrated because the voxel size of the laser beam is changed accordingly. The phase model employed for closed-loop control also requires re-calibration. Additionally, since the axial working distance of a high-NA objective lens is also reduced, the dispersion compensation unit, including the secondary objective lens and a lens pair, needs to be adjusted accordingly.

Regarding components and models used in the experiments, we have made the following updates in the manuscript to include them (revised parts in the manuscripts are highlighted in yellow):

- Add specific information of grating (G2) in line 4 of page 3 (main text): "After a mirror M5, lenses (L5, L6), and grating (G2, T-1000-1040s, LightSmyth)"
- Add XYZ stage model in line 4 of page 2 of the Supplementary Materials: "The sample (i.e., photoresists on a substrate) is mounted on a precision XYZ stage (L-511 for the z-axis; and L-509 for the x- and y-axis, Physik Instrumente) for large-scale TPL."

Comment 1-4:

The authors report an open-loop error of 1139 nm. This appears quite large. What factors contribute to such a high value, and is this measurement obtained from their own system or sourced from previous literature? The reported structures show curved edges rather than the sharp boundaries expected for a cuboid. The authors should clarify why the edges are rounded and whether this results from voxel shape, beam aberrations, or processing parameters. Additionally, if the writing speed is 100 mm/s for structures of 8×8×1 μm^3 and 8×8×4.8 μm^3 with a 100 nm hatch spacing, it is unclear why the total fabrication time is 12 hours. The slicing distance is also not specified, which is necessary to understand the total voxel count and actual writing duration.

Response 1-4: Thank you for your question. Regarding open-loop errors shown in Figure 1c, the results are from our own experimental data. The main reasons that open-loop errors were not discussed in the literature previously is that (1) typical demo structures are small in size (< 100s microns), and thus often have a short printing time (< 2-3 hours); and (2) people tend to report the best results with fine calibration (including our team). Yet, it is a common knowledge in the field of TPL that best writing conditions drift over time. And seeing a micron level drift is not surprising for a printing time of > 10 hours. The source of the errors, as discussed in Response 1-1 (and more in Response 3-1-1), can include drifting laser power, temperature, humidity, mechanical vibration, and motions of liquid resins etc. However, we'd like to point out that identifying and eliminating the error sources is not the focus of this work because once closed-loop control is implemented, all errors from different sources will be effectively compensated. Our key novelty lies in the fact that despite these complex adverse factors, this approach can autonomously fabricate target structures with low printing errors (< ± 15 nm) over extended durations.

Regarding the shape of cuboid, firstly, the voxel shape of a laser beam is ellipsoidal; as such, the printed edges cannot be perfectly sharp. Secondly, the line-scan measurement results represent the integral of light intensity distributions corresponding to different wavelengths across the laser spot. Specifically, the light spots at the structural edges exhibit an integral transition effect, which accounts for the non-steep profile observed in the measurement results.

Regarding the fabrication time, the cuboid array was printed repeatedly at one-hour intervals for a total duration of 12 hours. Both the cuboid and slabs are single voxel-layer structures;

therefore, no slicing distance is needed; the height of the structure is entirely determined by the laser doses, which further demonstrates the effectiveness of precise height and dose control.

Comment 1-5:

The reported average height errors for the 1.0-μm and 4.8-μm cuboids are 0.130 μm (s.d. 0.088 μm) and 0.084 μm (s.d. 0.069 μm), respectively. While the authors attribute these deviations to temperature fluctuations and mechanical vibrations, such factors can generally be minimized under controlled lab conditions using stable temperature/humidity regulation and pneumatic isolation tables. Laser power drift typically affects much larger structures, so its role in height variation at these small scales should be reconsidered or clarified. Clarity on how the closed-loop system actively drives the process toward the target fabrication values is insufficient in the current text and needs further explanation. How do the authors plan to control or compensate for beam distortions in real time?

Response 1-5: Thank you for your questions. As an international leading group in TPL technology, we are well aware of all kinds of possible factors that can influence the printing process, including all the factors that you mentioned previously; we also know how to eliminate these factors by fine-controlling the environment parameters (temperature, humidity, vibration etc.) and laser condition (power drift, beam profile change, etc.).

Yet, the purpose of this work is to achieve a much higher repeatability without doing any additional system/dose calibration, laser warm-up/control (to minimize power drift), or strict environmental control. The DCI module monitors the final part dimension, and therefore as long as it meets the control target, we do not need to worry about all other factors, including beam distortion. And our experimental results have proved our closed-loop solution has achieved substantially higher reproducibility than any previously reported work, especially for long printing sessions (> 10 hours) that drift will always occur regardless of any level of system calibration and environment control.

Comment 1-6:

References:

[1] Liu, Tianqi, Peipei Tao, Xiaolin Wang, Hongqing Wang, Minfei He, Qianqian Wang, Hao Cui et al. "Ultrahighprinting-speed photoresists for additive manufacturing." Nature Nanotechnology 19, no. 1 (2024): 51-57.

[2] Jiao, Binzhang, Fayu Chen, Yuncheng Liu, Xuhao Fan, Shaoqun Zeng, Qi Dong, Leimin Deng, Hui Gao, and Wei Xiong. "Acousto-optic scanning spatial-switching multiphoton lithography." International Journal of Extreme Manufacturing 5, no. 3 (2023): 035008.

Typographical errors:

1. Line 84: 40X should be mentioned as 40×

2. Figure caption 3: inconsistent usage of (), please follow the same usage throughout the aricle.

3. Figure caption 3: SEM image (a), AFM image (b),….. can be rewritten as: a. SEM image, b. AFM image…

Response 1-6: Thank you for your comment. We have corrected these errors in the revised manuscript (Fig. 3 etc.). All modified parts are highlighted in yellow.

References

[R1] Zvagelsky, R., Mayer, F., Beutel, D., Rockstuhl, C., Gomard, G., & Wegener, M. Towards in-situ diagnostics of multi-photon 3D laser printing using optical coherence tomography. Light Adv. Manuf. 3, 466-480 (2022).

[R2] He, Y., Shao, Q., Chen, S. C., & Zhou, R. Characterization of two-photon photopolymerization fabrication using high-speed optical diffraction tomography. Addit. Manuf. 60, 103293 (2022).

[R3] Zvagelsky, R., Kiefer, P., Weinacker, J., & Wegener, M. In-situ quantitative phase imaging during Multi-photon Laser Printing. ACS Photonics 10, 2901-2908 (2023).

Reviewer #2 (Remarks to the Author):

General comment:

The authors present a study on the development of a two-photon lithography technique integrating a smartly developed and integrated closed loop to measure in real time variations of the actual sample modified, providing high control over the final height resolution imprinted. The approach enables measuring a real-time phase and height maps that feed a closed-loop controller that iteratively adjust the printing parameters regarding with the laser pulse energy/dosage, scanning rate and exposure time. The error using the close loop system is always smaller over long processing times compared to that of operating the same system under an open loop configuration. These results apply to the specific case of the fabrication of a diffractive optical mask. The approach presents high height reproducibility and stability. I consider the paper can be accepted for publication in Nature Communications only after some minor clarifications from the authors regarding:

General response: Thank you for your overall positive comments. Following, we provide detailed responses to each of your comment.

Comment 2-1:

Specific calibration requirements for different polymer resins reduce compatibility with other systems. It is already highlighted that these results apply only to the photoresist IP-L 780. Additional comments should be added pointing to the parameters that need to be considered towards applicability of the current approach with other photoresists.

Response 2-1: Thank you for your comment. Indeed, although our team has developed different classes of custom-resins, we used a commercial photoresist IP-L 780 that most people in the field are familiar with as a representative photoresist in all experiments to demonstrate our method. In general, the closed-loop TPL method is applicable to all classes of two-photon resins, both custom-developed and commercial products.

Following are the requirements for resins to be used in our closed-loop TPL method: (1) the liquid photoresist needs to show a refractive index change between their uncured and cured states. This characteristic is inherent to all commercially available photoresists to date, such as IP-Dip and IP-S [R1, R2]; and (2) The printed part should have negligible volume change post development because our closed-loop method only monitors the photopolymerization process, i.e., from liquid resins to its cured solid state. It is worth to note that post development volume can change for some resin classes. This issue can be largely addressed by adjusting the printing power to a moderately high value within the reaction window. Alternatively, photoresists with a consistent polymer shrinkage ratio across different control parameters of our closed-loop system are also compatible with this approach. We have included the discussion here in the revised manuscript (Page 4, line 167). All modified parts in the revised manuscripts are highlighted in yellow.

Comment 2-2:

As lateral resolution is not increased, but on the contrary, it is worsened, the applications for which the current approach is limited to flat phase masks and plates to be later used as diffractive optical elements. As resolution increase from the optical point of view are feasible, the authors should comment on possible approaches that would increase lateral resolution.

Response 2-2: Thank you for your comment. Although the lateral resolution of the dual-comb interferometer (DCI) can be further optimized, it is still diffraction limited and will still not be sufficient for monitoring the TPL in-plane process, which typically has a minimum feature size of < 150 nm. For this reason, we did not try to optimize the lateral resolution of the DCI module, which can be done by using a high-NA (e.g. NA = 1.45), oil-immersion objective lens. We have revised the manuscript to include the discussion of increasing the lateral resolution (page 7, line 271-272).

Comment 2-3:

Data included in Figure 4c suggests that after 4 hrs, the open-loop configuration would not work anymore. It is not clearly stated why. Can the authors comment on that?

Response 2-3: Thank you for your question. Figure R2-1 below presents microscopic images of the post-processed structures printed over time. As can be observed in the third row, no structures were printed after 4 hours using identical printing parameters. We believe this occurred because the target part height (1 μm) is very small, making it susceptible to environmental perturbations (stage vibrations, drift etc.). Consequently, no two-photon polymerization reaction was initiated after 4 hours for the open-loop operation. In contrast, the

closed-loop system works fine (i.e., structures of different heights in the first and second rows). It is worth to note that most TPL papers printed small structures (<100s microns in size) for demonstration, which need short print time (< 1 hour) and thus did not see significant parameter drifting effect. To clarify the matter, we have included new figures (i.e., Supplementary Fig. 7) in the supplementary materials to reflect our explanation here. All modified parts in the revised manuscripts are highlighted in yellow.

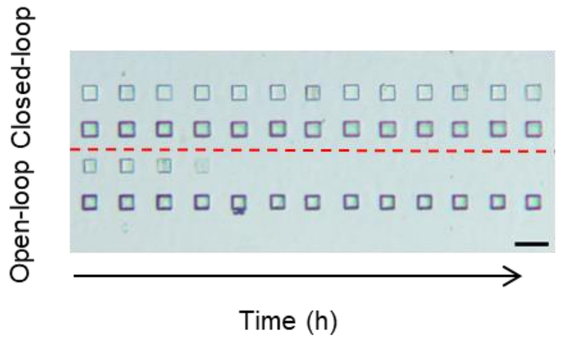

Figure R2-1 *Optical image of the post-processed cuboid structures in Fig. 4c,d. The control setpoints for the first and third rows are 1.0 μm; and those for the second and fourth rows are 4.8 μm. Scale bar: 20 μm.*

Comment 2-4:

From the experimental point of view, I wonder if the lack in lateral resolution comes from the fact that the beam is being focused by a regular lens and not by an F-theta lens. The beam is always entering at different positions because of the galvanometric scanner is scanning the beam before it enters the objective. Therefore, it will already produce a focal spot at different planes (or following a spherical surface). Can the authors include estimated variation given by this effect? On that line, would a high numerical aperture objective improve lateral resolution? The main challenges for using different optics (high NA for example) should be mentioned.

Response 2-4: Thank you for your comment. The lateral resolution shown in Figure 2a is the lateral resolution of the in-situ measurement system (i.e., DCI module) instead of the resolution of TPL printing system; the TPL system has a 100-nm writing resolution that the DCI module cannot resolve; and thus, we did not try to optimize the lateral resolution of the DCI system as discussed in Response 2-2. As the target application in this work is to fabricate precision phase plates, we did not emphasize the in-plane printing resolution of TPL, which is widely reported in different papers. The two-axis galvo was used for steering the laser beam in TPL printing. Therefore, the lateral resolution shown in the main text is not limited by the aberration from the scan lens. Additionally, we used a Thorlabs' telecentric scan lens (LSM04-BB, Thorlabs, Inc.) for laser scanning, which can offer minimal optical aberrations, low F-Theta distortion and flat image planes.

As mentioned, although a high-NA objective lens can improve lateral resolution of both the TPL system and the in-situ measurement system, we used a 40x, medium-NA objective in the

experiments for the following reasons: (1) to increase printing range (both laterally and axially); and (2) for printing phase plates, with closed-loop TPL, it does not compromise axial precision. We have revised the manuscript to include the discussion here.

Comment 2-5:

The nominal pulse duration in this case is in the hundreds of fs. There are many optical elements that may induce temporal dispersion on the femtosecond pulses, and when the pulses arrive at the irradiation region, they are surely longer in time. Is the pulse duration and the pulse dispersion in time a parameter that affects considerably the printing outcomes? Resin compatibility may be affected radically when the pulse is a lot longer than nominal values at the exit of the laser source. Additional comments should be added in that regard.

Response 2-5: Thank you for your comment. In fact, unlike the metrology arm, the fs laser in the scanning path did not pass too many optical elements (i.e., only a few high-reflectivity mirrors, galvo mirrors, and objective lens etc.); thus, the induced dispersion is small and will not affect the printing quality and required laser power; on the other hand, if the pulses are too widened, the negative effect would be obvious (i.e., incomplete polymerization and increased voxel size [R3]). To confirm our discussion, we measured the pulse width after the objective lens to be ~200 fs (via an autocorrelator), which is fine for TPL.

Comment 2-6:

Final comment is regarding the experimental details on the characterization of the Fresnel Zone Plate lenses. Although the experimental setup is included in the supplementary material, I find it lacks description of elements such as the incorporation of quarter wave plates in the characterization path, and the use of an additional balanced photodiode. This part is just for the characterization, but for the sake of completeness, additional comments should be included.

Response 2-6: Thank you for your comment. A detailed description of the characterization of the Fresnel zone plate lenses is provided on page 3 in the Supplementary Information, including the optical elements and the measurement setup. (See **Supplementary Figure 5**). Note that the quarter wave plates and balanced photodiode are part of the DCI module and were not used in FZP measurement setup. All modified parts in the revised manuscripts are highlighted in yellow.

References

[R1] Zvagelsky, R., Kiefer, P., Weinacker, J., & Wegener, M. In-situ quantitative phase imaging during Multi-photon Laser Printing. ACS Photonics 10, 2901-2908 (2023).

[R2] He, Y., Shao, Q., Chen, S. C., & Zhou, R. Characterization of two-photon photopolymerization fabrication using high-speed optical diffraction tomography. Addit.

Manuf. 60, 103293 (2022).

[R3] Tan B, Venkatakrishnan K, Makaronets A. Effects of pulsewidth on two-photon polymerization. Des. Monomers Polym. 16, 145-150 (2013).

Reviewer #3 (Remarks to the Author):

General comment:

This article presents a novel phase imaging technique to measure the change in phase that occurs during two photon lithography (TPL) and applies it to demonstrate higher-precision printing of optical elements, such as diffractive optical elements (DOE) and Fresnel zone plates (FZP). The improvement in precision was achieved through real-time feedback control, which has been challenging to achieve in TPL. Amongst the different types of functional materials that are produced by TPL (such as bio-scaffolds, mechanical metamaterials, etc.), optical elements tend to have more stringent precision requirements. Therefore, this work is timely and valuable for the practical implementation of TPL for industrial scale use. However, there are several issues with this study that require revision.

General response: Thank you for your overall positive comments. Following, we provide detailed responses to each of your comment.

Major issues:

Comment 3-1:

1. A clearer explanation of the premise of this study should be provided. In its current form, this article may be confusing to readers who are new to TPL. In general, voxel formation in TPL is remarkably repeatable. If the input parameters (such as laser power and scanning speed) are held constant, one expects to obtain repeatable voxel geometries. Lack of precision in large structures (i.e., assembly of voxels) usually arises from the inability to hold the process parameters constant (over a long period of time) or due to motion errors (i.e., errors in alignment, positioning, or speed).

Response 3-1: Indeed, as one of the leading TPL research groups, we totally agree with your comment; and holding the process parameters is not difficult for short printing sessions (< 2 hours) and that's why we see many amazing SEM images of fine-TPL structures. We have revised the manuscript to make this message clear to our readers (page 2, line 50-51). (All modified parts in the revised manuscripts are highlighted in yellow.)

Owing to the limited TPL printing rate, printing large parts is time-consuming (>10 hours), and thus holding the process parameters become extremely challenging, which is the main problem we are solving in this work; in addition, to fabricate fine-structures (< 200-nm line width), it usually requires fine calibration of laser doses; our work further eliminates the need for power calibration and laser warm-up etc., making high-precision TPL a turn-key solution.

Comment 3-1-1:

1.1. The authors have not specified the sources of error that are being compensated here. Are they compensating for motion errors (such as XYZ stage drifting away from its set point height, a pre-existing tilt of the substrate, or some other error motion) or errors due to variation in the laser power over time? Can the authors specify the error sources being compensated here and support their claims with relevant data, such as laser power drift over time, position of the Z stage over time, etc.?

Response 3-1-1: Thank you for your comment. Traditionally, to print 100-nm level structures via TPL, all environmental factors (including temperature, humidity, mechanical/ground vibration etc.) and system stability factors (including laser power, beam profile, pulse width drift; galvo-scanner vibration/jittering etc.) need to be well controlled. This is not only very expensive, but also time consuming (e.g., warming up a femtosecond laser amplifier to stable states takes a few hours, which is what we did in our previous TPL work, Saha et al. *Science*, 366(6461): 105-109, 2019.)

The purpose and value of the closed-loop TPL solution is to achieve a substantially improved reproducibility without the need to compensate for different environmental and system factors. **This is feasible because the DCI module directly monitors the final part height and immediately feeds the measured data to the controller and sets new laser exposure parameters at 360 Hz instead of simultaneously controlling many different environmental and system factors.** In other words, although laser power drift or vibration can change the voxel size or position, the designed part will always be made with the target dimension and precision.

Below we provide more discussions on possible sources of fabrication errors in open-loop TPL printing, which become no longer important after the introduction of closed-loop TPL. Firstly, regarding laser power: during the comparison experiments between open-loop and closed-loop TPL fabrication in Fig. 4c and 4d, we recorded the power variation of the 800-nm laser for printing, as illustrated in Fig. S1. Over a 12-hour period, the laser power fluctuation was less than 1 mW. Secondly, concerning the motorized displacement stage: the Z-axis stage is PI L-511.2ASD00, featuring a repeatability of 150 nm and direct position measurement. In open-loop printing, the Z-axis position remains unchanged, and thus it does not contribute to the open-loop errors observed in Fig. 4c and 4d. Finally, regarding the tilt of the sample-carrying platform: prior to the TPL printing, we calibrated the platform's tilt via elevation angle adjustments. The specific procedure involves, after selecting the printing area (e.g., a 3 mm × 3 mm rectangular region), conducting printing tests with identical parameters near the four corners and adjusting the x- and y-direction tilt knobs to achieve consistent curing heights, at

which point the plane of the region is considered orthogonal to the incident direction of the printing laser.

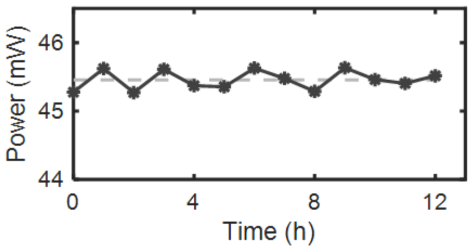

Figure S1 Measured power of the 800-nm fs laser monitored during the open-loop test shown in Fig. 4 in the manuscript.

Another important open-loop error is substrate unevenness. In the printing tests for anchorage, the initial position of the printing laser focus is set 1 µm below the substrate surface. When printing different pixels, only the x and y stage positions are moved, and the substrate's unevenness affects the relative position of the printing focus to the substrate surface, leading to inconsistent losses in printing laser power. Therefore, the curing height is highly sensitive to the substrate surface unevenness. This explains the errors seen in the open-loop cuboid printing tests in Fig. 1c. The substrates involved in our work include silicon and glass. For silicon wafers, according to SEMI M1 standards, the typical maximum values for total thickness variation (TTV), bow, and Warp in 100 mm polished single crystal silicon wafers are 10 µm, 40 µm, and 40 µm. Previous experiments have measured bowing and warping levels at micron level in silicon wafers. [R1] The printing results presented in the main text, including Fig. 4c and 4d, were performed on a 10 mm × 10 mm × 1 mm glass substrate to fabricate transmission-type DOE and FZP. The glass substrate is bonded to a slide fixed on the stage, which complies with the ISO 8037-2:1997 product standard, i.e., the maximum acceptable non-flatness is 25 µm for 46 mm length. Although data in [R2] indicate that glass substrates can achieve flatness comparable to silicon substrates, with TTV less than 2 µm and warp less than 30 µm for 8-inch glass wafers, glass has a lower Young's modulus than silicon [R3].

In the 10+ hours open-loop printing tests, the results show similar nonlinear trends. They stabilize slowly after ~hours. This can be seen in Fig. 1c, 4c, and 4d. We believe this happens because the photoresist's light-sensitive properties change. The change comes from long exposure to the test environment. At the start of these three tests, we took the photoresist fresh from a strict storage setup. After sufficient air exposure, its photosensitivity was gradually weakened and eventually stabilized. Previously, TPL printing was mostly conducted in a short period of time (< 2 hours) to fabricate parts around 100 micron in size for SEM measurement and demonstration. Yet, long printing time (> 10 hours) is required to fabricate large parts for practical applications. In this case, closed-loop TPL can fundamentally address the challenge regardless of different external factors.

References:

[R1] Park, J., Bae, J., Jang, Y. S., & Jin, J. (2020). A novel method for simultaneous measurement of thickness, refractive index, bow, and warp of a large silicon wafer using a

spectral-domain interferometer. Metrologia, 57(6), 064001.

[R2] https://www.schott.com/en-gb/expertise/applications/glass-carrier

[R3] Trott, Gary R., and Aric Shorey. Glass wafer mechanical properties: A comparison to silicon. 2011 6th International Microsystems, Packaging, Assembly and Circuits Technology Conference (IMPACT). IEEE, 2011.

Comment 3-1-2:

1.2. From Figure 4c, it appears that the error being compensated is the tilt of the substrate. If so, would it not be easier to measure the tilt of the stage before printing and then adding this as a compensation term to the commanded Z location of the stage? Can the authors compare the tradeoffs of their approach and the tilt compensation approach?

Response 3-1-2: Thank you for your question. Before the experiment, the level of the substrate/sample stage has been carefully calibrated and thus substrate tilt is not an error source. Please refer to Response 3-1-1 for detailed calibration processes and error source analyses.

Comment 3-1-3:

1.3. The compensation approach seems to rely on increasing the amount of printing (by increasing the number of pulses) so that more printing can occur in regions where the focal plane has moved away from the substrate (and thus has a lower printed height). Would this approach work well in other scenarios, such as those listed in the introduction (vibration, and laser power fluctuations), particularly with respect to the time scales of these other error sources.

Response 3-1-3: Thank you for your comment. Yes, our method works for other scenarios as the DCI measures and controls the final part height regardless of different sources of errors. Specifically, the DCI module in-situ measures the phase values and subsequently makes compensation decisions, without imposing restrictions on the compensation methods, such as adjustments to pulse number (exposure time), scanning speed, laser power, or Z-location. In the closed-loop printing demonstrated in this manuscript, we chose to modulate the pulse number and employ repeated exposures to achieve the compensation.

On the temporal scale, compensation for long-term errors has been demonstrated in this work. Influences from error sources on shorter timescales (less than 1 s) were not prominently observed in our system; however, our method is theoretically capable of compensating for them. The detection frequency of the DCI module is 360 Hz, and parallel measurements are feasible (demonstrated in our previous work in [R4]), though faster compensation would necessitate consideration of the time costs associated with data processing and the control program.

References:

[R4] Lu, W. et al., "Scan-less 3D microscopy based on spatiotemporal encoding on a single-cavity dual-comb laser," *Optics Letter*, 49, 1766-1769 (2024).

Comment 3-1-4:

1.4. Related to the point above, increasing the amount of printing will not only increase the height of the structures but also the width of the structures (and thereby introduce errors). What is the typical deviation of the measured width from the designed width of the blocks (during close-loop operation)? The error introduced along the lateral direction may not be an issue for DOE and FZP structures, as the optical performance is more sensitive to height errors. However, for other applications, this may become a limitation. Can the authors comment on the generalizability of their compensation approach in light of this limitation?

Response 3-1-4: Thank you for your comment. Indeed, when printing a single-voxel line/wire, increasing the laser dose would increase both the width and height of the part. This increase is owing to the increased voxel size when laser doses increase. Notably, the increase in size in the lateral and axial directions is following a 1:2 ratio, which is the ratio between the lateral and axial dimension of a Gaussian focus. Using this relationship, the precise axial measurement and closed-loop control can potentially be extended to fine-controlling the lateral dimension of a TPL printed part. Once again, our method is most effective for the precise fabrication of phase plate type devices (e.g., DOE and FZP), which has a wide range of applications.

Comment 3-1-5:

1.5. The authors have not provided any comparison of the quality of their optical structures (DOE or FZP) with respect to those printed by others (for example Reference 21). Did past researchers experience same errors and how did they solve it? Now that the authors have a new control strategy, what improvements can the readers expect over the state-of-art of DOE and FZP fabrication?

Response 3-1-5: Thank you for your question. Compared to other state-of-the-art TPL results of DOEs or FZPs, our method has achieved comparable resolution and quality (with improved reproducibility). It is worth to note that most TPL papers only emphasize their improved resolution or speed; and thus, most of the work did not fabricate large parts (or many identical small parts over a long period of time) that would require long printing time and try to discuss the reproducibility challenge. In the past, such challenge can only be addressed by fine-controlling the environment and laser system; best results are often achieved via trial-and-error experiments over time. Although our method does not directly improve resolution, it substantially improves the reproducibility to ±15 nm without the need of environment and laser control and calibration, which makes TPL suitable for industry applications. Previous studies rarely address the reproducibility problem because as long as the results can be repeated, the scientific community would acknowledge the results (e.g., new records on resolution and rate).

Comment 3-2:

2. A major assumption in this work is that the change in refractive index (RI) of the resist due

to curing is constant (Equation 3). However, it is well-known that the change in RI depends on the dosage during TPL printing (for example, see: https://doi.org/10.1364/OL.44.000029). For large dosages, such as in the lower-right quadrant of Figure 2(d), the authors' approximation may be reasonable. However, is Equation 3 still valid at low dosages, such as in the upper-left quadrant of Figure 2(d)? For example, could the authors provide a comparison of measured phase versus measured height (such as Figure 2(e)) for low dosage conditions? Is the trend still linear? Related to this, what was the power for the data shown in Figure 2(e)?

Response 3-2: Thank you for your comment and question. Your concern is valid as laser doses can indeed change the RI of a cured part slightly. In the TPL printing process, parameters such as laser power, galvo scanning speed, and pulse number (i.e., exposure time controlled by the AOM in our system) can all lead to small RI variations, which are attributed to differences in exposure doses, as reported in prior studies [R5, R6].

Despite the abovementioned issue, our method is still valid and accurate for the following reasons: (1) the induced RI variation due to laser dose differences is small; and (2) we have carefully calibrated the RI variation induced by photopolymerization as shown in Fig. 2e (i.e., $\Delta n \cdot 2\pi/\lambda_0$). The measurements in Figure 2e were obtained by varying the pulse number under constant laser power to modulate the curing height; thus, the resulting fit inherently incorporates refractive index variations arising from exposure dose (i.e., pulse number). Our experiments demonstrate that deriving Δn in Equation 3 from these fitted results, and subsequently converting it to the phase distribution for DOEs or FZPs (in an air medium), is feasible. Note that RI variations are precisely monitored via our custom-built optical diffraction tomography system during the calibration process (He et al., "Characterization of two-photon photopolymerization fabrication using high-speed optical diffraction tomography," *Additive Manufacturing*, Vol. 60, pp.103293, 2022.).

On the other hand, low laser power and doses were not used in all our experiments because cured part may suffer from volume change at low laser doses, which generate dimensional errors that cannot be compensated by the closed-loop TPL solution. For more discussions on this issue, please find them in Reviewer 2's **Response 2-1**.

References:

[R5] Dottermusch, S. et al. Exposure-dependent refractive index of Nanoscribe IP-Dip photoresist layers. Opt. Lett. 44, 29-32 (2019)

[R6] Zvagelsky, R., Mayer, F., Beutel, D., Rockstuhl, C., Gomard, G., & Wegener, M. Towards in-situ diagnostics of multi-photon 3D laser printing using optical coherence tomography. Light Adv. Manuf. 3, 466-480 (2022).

Comment 3-3:

3. The authors have used the number of pulses as the controllable parameter in this study, but it has not been defined. If the beam is not scanned, the interpretation is straightforward (as it is determined by the duration of exposure at any given location). However, what is the interpretation of this parameter when the beam is being scanned? Is it the number of pulses that

are incident on any given material point (in which case it cannot be tuned without changing the scan speed) or is it the total number of pulses incident over a scanned area? If it is the later, it suggests that the same region is being scanned multiple times and changing the duration of exposure implies changing the number of times the same region is being scanned. If so, this should be specified in the text.

Response 3-3: Thank you for your question. Since the laser repetition rate is extremely high (80 MHz) in reference to the slow scanning speed, it is reasonable to assume that the neighboring laser pulses arrive at the same spot. To investigate the effective phase difference induced by TPL in the photoresist in Fig 2c, we primarily varied the laser power and the number of pulses in the printing system. Here, the number of pulses refers to the average number received per curing point during scanning, which is determined by AOM modulation. Notably, in all experiments, a consistent scanning rate of 30 mm/s was used; thus, the laser dose is governed by the laser power and the AOM activation time only (i.e., corresponding to the number of pulses). For digital control, the printed image is first discretized to obtain a point array representing the entire scanning path. The average time T_{galvo} at each scanning point is determined by the scanning rate and remains constant throughout this work. Therefore, within each T_{galvo}, controlling the AOM activation time $T_{\text{AOM\_ON}}$ determines the number of pulses at each scanning point. For example, a pulse number of 5000 corresponds to a $T_{\text{AOM\_ON}}$ of 62.5 ms. To clarify the matter, we have revised the manuscript to include the discussion here (page 4, line 134-136). (All modified parts in the revised manuscripts are highlighted in yellow.)

Comment 3-4:

4. Can the authors clarify how the scanning of the imaging laser occurs? As the imaging laser forms a line illumination (and sensing occurs via a photodetector), presumably the laser should be scanned to generate 2D images such as those shown in Figure 3(c). However, it is not passing through the Galvo (laser through L4-M4-DM). How is the scanning achieved?

Response 3-4: Thank you for your question. In the experiments, the 2D phase map was obtained via line scanning, which was implemented by translating the stage orthogonally to the 1052-nm focal line, where different spectral components are dispersed and focused on this line. Considering the theoretical lateral resolution, the step size for the linear stage in Fig. 3c is set to 0.5 μm, and a single dual-comb measurement yields the phase distribution over an approximately 40-μm range along the focal line. We have revised the manuscript to include the discussion here (on page 5, line 159-160).

Comment 3-5:

5. Can the authors clarify how the scanning of the printing laser (focal point) and the imaging laser (focal line) are synchronized in space and time and its impact on averaging of phase information? Depending on the scanning paths and the scan rates, the phase information captured by the detector can come from various different locations on the sample and from various instants of time. For the structures demonstrated here, this may not be an issue because

the target geometry looks uniform over each 8 μm×8 μm slab. However, for more complex 3D geometries, this may become a limitation for real-time control. Can the authors comment on this?

Response 3-5: Thank you for your question. As shown in the phase imaging result in Fig. 3c, the 2D phase map was obtained by mechanically scanning the sample (via a translation stage) in a direction orthogonal to the 1052-nm metrology focal line (spectral components dispersed along this line; and each spectral component records phase variation at different positions along the line). During the closed-loop printing process, the metrology focal line is positioned at the center of the work field and remains continuously active, i.e., phase monitoring at 360 Hz; the timing of the phase information measurement and extraction as well as the fabrication laser (800-nm) is controlled and synchronized by the closed-loop control program. During fabrication, the metrology focal line is aligned with 800-nm fabrication laser focus's scanning direction (the galvo's fast scanning axis); and the galvo's secondary scanning axis actively aligns the printing line and the metrology focal line so that throughout the fabrication process, all voxels in the work field are continuously monitored. Based on this principle, more complex geometries can also be precisely made with closed-loop control without a problem.

Notably, we can potentially upgrade TPL system to a line-scanning configuration [R7], recently developed by our team, which will perfectly match the line-metrology DCI design with improved printing rate.

References:

[R7] Q. Zhong, C. Datar, W. Liu, G. Liu, X. Guo, X. Fan, F. Han, B. Chen, S. Gu, and S. Chen, "Full-bandwidth, Continuous, and Grayscale 3D Nanolithography via Line-illumination Temporal Focusing of Ultrafast lasers," arXiv preprint arXiv:2512.22746, 2025.

Comment 3-6:

6. What evidence can the authors provide in support of their claim in the Discussion section (lines 267-268) that their technique can be applied to control the lateral dimension of a designed part? As the phase measurement (Equation 3) is independent of the lateral geometry, it is difficult to appreciate how this technique can be applied to control lateral dimensions.

Response 3-6: We thank the Reviewer for this comment.

Thank you for your question. For parts with lateral dimensions above the diffraction limited (> 500 nm - μm), the DCI module can monitor the printing process in situ with sufficient precision; for sub-diffraction limit lateral dimension control, the control rationale is discussed in **Response 3-1-4**, i.e., as the voxel aspect ratio is fixed at 1:2, we can use this relationship to predict the lateral dimension. Note that conventionally (open-loop TPL), both lateral and axial dimension control is done with trial and error experiments and precise laser dose control.

Minor issues:

Comment 3-7:

7. In the abstract, the authors claim that laser power, scanning rate, and exposure time are being modulated. However, data for power and scanning rate modulation has not been presented. The authors may consider revising the abstract so that it is consistent with their work.

Response 3-7: We thank the Reviewer for this comment.

Thank you for your suggestion; we have revised the abstract as follows: lines 16-18: "*The measured phase information is fed to a dynamic model for modulating the printing parameters ~~(including laser power, scanning rate, and exposure time)~~ to correct height errors in a fully automated fashion.*" (All modified parts in the revised manuscripts are highlighted in yellow.)

Comment 3-8:

8. In the methods section (lines 296 -297), it is difficult to understand how the spectrum before TPL was obtained. This is because the optical elements for this measurement have not been shown in the main Figure 1 (but are shown later in the supplementary Figure 1). The authors may consider highlighting in the methods section that additional details on the optical setup are shown in the supplementary Figure 1.

Response 3-8: Thank you for your suggestion. Actually, we have presented the optical setup of the in-situ transmission measurement in Fig. 1 (see the optical path indicated by the light pink arrow). We have also described the design of this unit in lines 85–96 of the main text. And at the end of the same paragraph (line 99 – 100), we have mentioned more details are provided in Supplementary Fig. 1 and the Supplementary Methods section.

Comment 3-9:

9. Figures 6 (b) and (d) seem to have incorrect scale bars. The labeled lengths are inconsistent with the scale bar.

Response 3-9: Thank you for your suggestion. We have revised manuscript to correct the errors in Fig. 6 (b) and (d) and lines 263-264 accordingly. In addition, we also updated the figure caption in Supplementary Fig. 4. (All modified parts in the revised manuscripts are highlighted in yellow.)

Comment 3-10:

10. In the Introduction section (lines 36-37), the authors mention that in-situ imaging techniques are limited by diffraction. The authors may consider clarifying that the resolution of their technique is also limited by diffraction along the lateral dimension, but that it is not diffraction-limited along the depth direction due to its interferometric phase measurement mechanism.

Response 3-10: Thank you for your comment. The mentioned section (lines 36–37) was intended to explain the limitations of the epi-illumination microscopic unit for in-situ measurement in TPL in the introduction section; since it is mentioned before the introduction of the DCI design, it should not confuse readers. By the way, Fig. 2(a) and 2(b) report our lateral resolution and depth sensitivity, which would clarify the matter the reviewer mentioned.

Comment 3-11:

11. Consider using different marker styles for open versus closed loop in Figures 4(c) and (d).

Response 3-11: Thank you for your suggestion. We have updated the marker style in Fig. 4(c) and (d) as suggested.

Point-by-point response to reviewers' comments:

Reviewer #2 (Remarks to the Author):

The authors have incorporated and answered all the concerns, major and minor modificaitons proposed during the first round of reviews.

It has to be noted that additional data is now included into the new version of the paper, and that experimental details that previously were not reported are now included in full. In addition, new descriptions and figures were included, and additional discussion was added.

I consider the paper can be now accepted for publication in Nature Communications, and congratulate the authors for the nice work.

Response: Thank you for recognizing our efforts and helping to improve the manuscript.

Reviewer #3 (Remarks to the Author):

General comment:

For the most part, the authors have provided satisfactory responses. However, the authors' unwillingness to appropriately revise their manuscript to clarify the scope of their approach is a cause for concern.

General response: Thank you for recognizing our efforts in addressing your concerns and improving the manuscript. Below, we provide further revision and explanation to fully address your concern.

Comment 3-1:

1. The authors continue to claim in their response that "we'd like to point out that identifying and eliminating the error sources is not the focus of this work because once closed-loop control is implemented, all errors from different sources will be effectively compensated." Claiming that all errors will be compensated is a very broad claim and it is not accurate. If the stages drift, their technique will not compensate for this error. For example, consider the target structure being a woodpile porous 3D structure with voxel-level nanowires (i.e., ~300 nm wide wires with 1000 nm pitch). If the non-repeatability in such a structure is due to drift in Z-motion stage or vibration in the galvo, their compensation technique will not be effective. The authors explain in their response that they believe that the errors that they are compensating arise due to changes in the response of the resist over time or due to non-flatness of the substrate. The authors should consider explicitly mentioning these potential sources in their manuscript (either in the main text or the supplementary material). Based on this, they should also consider grouping potential error sources into those that can be compensated by their technique and those that cannot be readily compensated by their technique. Being forthright about the capabilities and limitations of their work will not diminish the value of their work.

Response 3-1: Thank you for your comment. Sorry about the misunderstanding in our last response, which is quoted below in full:

"*The source of the errors, as discussed in Response 1-1 (and more in Response 3-1-1), can include drifting laser power, temperature, humidity, mechanical vibration, and motions of liquid resins etc. However, we'd like to point out that identifying and eliminating the error sources is not the focus of this work because once closed-loop control is implemented, all errors from different sources will be effectively compensated.*"

We like to clarify that in the paragraph above, we did not intend to suggest at all that an uncalibrated TPL system with low-quality parts (e.g., XYZ stage) can achieve high repeatability using our closed-loop approach. In fact, without highly repeatable and precise components, e.g., XYZ stages and galvo scanners, and a vibration isolation table, none of the errors can be compensated. However, these components are almost a default TPL setup for people who try to print fine structures (< 200 nm) through fine calibration. Most of the time, even when they achieve high precision (open loop), the optimal condition cannot be maintained for too long (e.g., a few hours) owing to parameter drifting. It is under this typical condition, our closed-loop solution can quickly identify the optimal condition without complex calibration and maintain the optimal condition indefinitely until the end of the fabrication.

As such, our closed-loop TPL method can compensate errors with the following two characteristics: <u>(1) errors generated at a low-frequency range</u> (e.g., laser power drift); and <u>(2) errors that cause part height variation</u> (e.g., over/under laser exposure). Any error sources that meet these two conditions can be effectively compensated using our method. We have included the discussions here in the main manuscript to avoid any possible confusions.

Below, we summarize several representative error sources observed in our experiments (not an exhaustive list) that can be corrected by our method:

- Substrate warpage and surface unevenness, especially deformation at the hundred-nanometer scale and larger.
- Photoresist stability issues, including photoresist (fluid) motion on the substrate and volatility under ambient air conditions.
- Fluctuations/variations in ambient operating temperature and humidity.
- Laser output intensity instability.

These discussions have been included in the revised manuscript and Supplementary Information. (Modified parts are highlighted in yellow.)

Comment 3-2:

2. In their revision, the authors claim that the voxel aspect ratio is fixed at 1:2. This is not universally true in TPL as the aspect ratio can vary over a large range such as 1:1 to 1:6. The authors should provide evidence to support this claim or consider revising this argument based on the state-of-art of TPL.

Response 3-2: Thank you for your comment. Indeed, the voxel aspect ratio can vary over different printing conditions, including exposure doses, scanning rate, laser pulse width, and scanning strategy (e.g. point scanning vs. projection). It is also important to maintain a fixed voxel aspect ratio during fabrication to ensure consistent and uniform printed structures. This is achieved in our experiments/system by maintaining a consistent laser power and exposure dose through the closed-loop system.

Below, we characterized the voxel aspect ratio via SEM by printing suspended wires using the same parameters as in all experiments. As shown in Fig. S1b, the 0° view (top view) shows that the suspended wire has a measured lateral dimension of 150 nm. Next, in a 30° tilted view in Fig. S1c, the suspended wire was measured to have a projected width of 150 nm, which corresponds to an axial dimension of 300 nm. These results indicate the voxel aspect ratio is approximately 1:2 under our experimental conditions.

We have included the new results in the revised Supplementary Information. (All revised parts are highlighted in yellow).

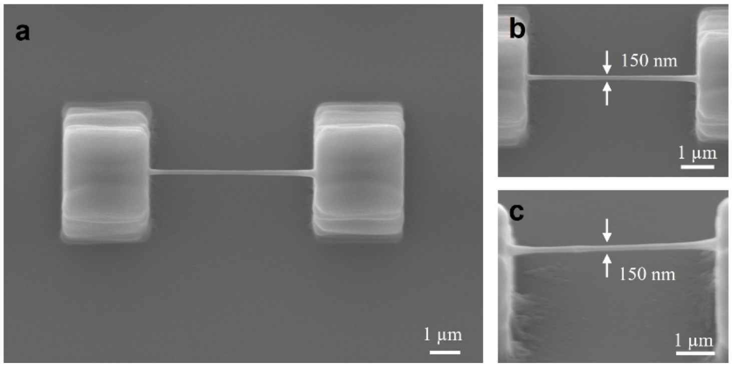

Fig. S1 | SEM images of the suspended-wire structure. (a) SEM image of the entire nanowire structure, supported by two bases. (b) Suspended wire with 0° stage tilt (top view). (c) Suspended wire with 30° stage tilt.
